# Supplementary material for: Discharge policies and care practices for children with suspected sepsis: A health facility scan at a nationally representative sample of hospitals and health centres in Uganda
Source: PLOS Glob Public Health. 2025 Aug 22;5(8):e0003559. doi: 10.1371/journal.pgph.0003559 (PMC12373204; doi:10.1371/journal.pgph.0003559)

**Discharge policies and care practices for children with suspected sepsis: A health facility scan at a nationally representative sample of hospitals and health centres in Uganda**

**Supplementary Material**

Table of Contents

[Table S1: Details of facility sampling and Facility Scan 2](#_Toc205995025)

[Table A. District-level and National-level Sample Selection 2](#_Toc205995026)

[5-survey environmental scan 2](#_Toc205995027)

[Table 1b. Facility Scan components and recruitment summary 3](#_Toc205995028)

[Table S2. Observed health worker care practices at admission 4](#_Toc205995029)

[Table S3. Observed vs reported discharge practices by service delivery level 5](#_Toc205995030)

[Table S4. Caregiver and health worker satisfaction with discharge care. 6](#_Toc205995031)

[Figure S1. Availability of facility resources by service delivery level and ownership type, n=36 facilities 8](#_Toc205995032)

[Figure S2a-e: Observed health worker care practices at admission, n=180 observations 9](#_Toc205995033)

[Figure S2a: Medical history taking: Asked and documented 9](#_Toc205995034)

[Figure S2b: Socio-economic history taking: Asked and documented 10](#_Toc205995035)

[Figure S2c: Physical measures: Performed and documented 10](#_Toc205995036)

[Figure S2d: Clinician observed measures: Performed and documented 11](#_Toc205995037)

[Figure S2e: Admission consultation topics discussed with caregiver 11](#_Toc205995038)

# Table S1: Details of facility sampling and Facility Scan

We used both district- and national-level sampling frames to capture a representative sample of the Ugandan health system’s diverse structure and to ensure balanced comparisons across service delivery levels and ownership types. For lower-level health facilities, we selected three hospitals and 13 health centres from two districts in Northern and Western Uganda, chosen for their demographic similarities, including a high proportion of children under five, comparable health care access, and varied health care infrastructure (Table 1) [19]. This district-level sampling allowed for a focused examination of local health care contexts with shared population characteristics. In contrast, to capture the variability of hospitals across Uganda, we used a national-level sampling frame to select 20 hospitals from 20 districts. This broader sampling approach ensured that our hospital sample reflected a wide range of geographical and operational contexts across the country. Study sites include both public and PNFP ownership types, representing both rural and urban geographical settings. Facilities were eligible for inclusion if they provided pediatric inpatient care and had no prior involvement in the Smart Discharges research program [20].

## Table A1. District-level and National-level Sample Selection

| **Service Delivery Level** | **District Sampling (n=16)** | | **National Sampling (n=20)** | | **Total**  **(N=36)** | |
| --- | --- | --- | --- | --- | --- | --- |
|  | **Public** | **PNFP** | **Public** | **PNFP** | **Public** | **PNFP** |
| Health Centre III | 10 (62.5) | 2 (12.5) | 0 (0.0) | 0 (0.0) | 10 (27.8) | 2 (5.6) |
| Health Centre IV | 1 (6.3) | 0 (0.0) | 0 (0.0) | 0 (0.0) | 1 (2.8) | 0 (0.0) |
| General Hospital | 0 (0.0) | 1 (6.3) | 10 (50.0) | 4 (20.0) | 10 (27.8) | 5 (13.9) |
| Referral Hospital | 0 (0.0) | 1 (6.3) | 5 (25.0) | 0 (0.0) | 5 (13.9) | 1 (2.8) |
| Regional Referral Hospital | 1 (6.3) | 0 (0.0) | 0 (0.0) | 0 (0.0) | 1 (2.8) | 0 (0.0) |
| National Referral Hospital | 0 (0.0) | 0 (0.0) | 1 (5.0) | 0 (0.0) | 1 (2.8) | 0 (0.0) |
| Total | 12 (75.0) | 4 (25.0) | 16 (80.0) | 4 (20.0) | 28 (77.8) | 8 (22.2) |

## 5-survey environmental scan

All study sites underwent a 5-survey Facility Scan informed by the Pediatric Sepsis Data CoLaboratory’s (Sepsis CoLab) Environmental Scan [21] which is used to support health facilities in identifying quality improvement priorities in treating children with sepsis [22]. These five surveys are comprised of i) a Scan to assess facility resources; ii) a Scan to assess a facility’s level of technological preparedness; iii) a Scan to assess the quality and safety of care through observation of a health worker in suspected or confirmed cases of severe illness/infection; iv) a Caregiver Satisfaction Questionnaire to assess patient-caregiver perceptions of the quality of care received at the facility; and v) a Health Worker Satisfaction Questionnaire to assess health worker perceptions of the quality of care provided a the facility [21]. The Environmental Scan was developed by 12 emergency medicine and critical illness experts in LMICs [22] and piloted in six African hospitals and demonstrated feasibility for assessing priority areas to enhance quality of care for critically ill children in low-resource settings [23].

Each of the five Sepsis CoLab Environmental Scans were enhanced to address specific areas (including discharge planning, post-discharge counselling, provision of take-home material, and follow-up referrals) relevant to discharge care through consultations with the local study team members which included pediatric acute and critical care investigators and health workers. We also omitted the non-discharge relevant aspects of the original scans. The discharge modules designed for this Facility Scan have since been incorporated in the Sepsis CoLab’s Environmental Scan, which is publicly available via Borealis, the Canadian Dataverse Repository [21].

## Table A2. Facility Scan components and recruitment summary

| Survey tool | Unit of focus | Number completed | Consent |
| --- | --- | --- | --- |
| **Facility Resources Survey** | Facility | 36 | Waived |
| **Facility Technological Preparedness Survey** | Facility | 36 | Waived |
| **Patient Observational Scan** | Patient | 180 (5 per facility) | Waived |
| **Caregiver Satisfaction Survey** | Caregiver | 180 (5 per facility) | Informed consent |
| **Health worker Satisfaction Survey** | Health worker | 180 (5 per facility) | Informed consent |

# Table B. Observed vs reported discharge practices by service delivery level

|  | **Hospitals (n=115 case observations)** | | | | | | | | **Health Centres (n=65 case observations)** | | | | | | | |
| --- | --- | --- | --- | --- | --- | --- | --- | --- | --- | --- | --- | --- | --- | --- | --- | --- |
|  | **Observed/ reported** | | **Observed/not reported** | | **Not observed/ reported** | | **Not observed/ not reported** | | **Observed/ reported** | | **Observed/ not reported** | | **Not observed/ reported** | | **Not observed/ not reported** | |
|  | **n** | **%** | **n** | **%** | **n** | **%** | **n** | **%** | **n** | **%** | **n** | **%** | **n** | **%** | **n** | **%** |
| **Providing discharge education** | 58 | 50.4 | 7 | 6.1 | 42 | 36.5 | 8 | 7.0 | 43 | 66.2 | 5 | 7.7 | 7 | 10.8 | 10 | 15.4 |
| **Providing take-home materials** | 41 | 35.7 | 0 | 0.0 | 54 | 47.0 | 20 | 17.4 | 4 | 6.2 | 0 | 0.0 | 11 | 16.9 | 50 | 76.9 |
| **Conducting post-discharge risk assessment** | 38 | 33.0 | 8 | 7.0 | 32 | 27.8 | 37 | 32.2 | 1 | 1.5 | 0 | 0.0 | 39 | 60.0 | 25 | 38.5 |
| **Scheduling follow-up referrals*** | 37 | 32.2 | 7 | 6.1 | 38 | 33.0 | 33 | 28.7 | 0^a^ | 0.0 | 12 | 18.8 | 5 | 7.8 | 47 | 73.4 |
| ^*^ 1 health center had missing data, total n=64 for this row among health centers | | | | | | | | | | | | | | | | |

# Table C. Observed health worker care practices at admission

|  | **Hospitals (n=115)** | | **Health Centres (n=65)** | | **All (n=180)** | |  |
| --- | --- | --- | --- | --- | --- | --- | --- |
|  | **n** | **%** | **n** | **%** | **n** | **%** | **p-value** |
| **Medical history taking: Asked and documented** |  |  |  |  |  |  |  |
| Age or date of birth | 115 | 100.0 | 33 | 50.8 | 148 | 82.2 | <0.001 |
| Symptoms | 113 | 98.3 | 63 | 96.9 | 176 | 97.8 | 0.621 |
| Duration of illness | 82 | 71.3 | 57 | 87.7 | 139 | 77.2 | 0.020 |
| History of illness | 65 | 56.5 | 54 | 83.1 | 119 | 66.1 | <0.001 |
| Feeding | 47^a^ | 41.6 | 41^b^ | 64.1 | 88^c^ | 49.7 | 0.007 |
| Recent antibiotic treatment | 33^d^ | 28.9 | 35 | 53.8 | 68^e^ | 38.0 | 0.001 |
| Immunization history | 27 | 23.5 | 25 | 38.5 | 52 | 28.9 | 0.050 |
| 7-item medical history scores, Median (IQR) | 4 | 3 - 5 | 5 | 4 - 6 | 4 | 3 - 6 | 0.008 |
| **Socio-economic history taking: Asked and documented** |  |  |  |  |  |  |  |
| Bed net use | 6 | 5.2 | 28 | 43.1 | 34 | 18.9 | <0.001 |
| Number of children in the family | 7 | 6.1 | 13 | 20.0 | 20 | 11.1 | 0.069 |
| Boiling or disinfecting drinking water | 3 | 2.6 | 16 | 24.6 | 19 | 10.6 | <0.001 |
| Sibling death | 2 | 1.7 | 2 | 3.1 | 4 | 2.2 | 1 |
| Access to pit/latrine/toilet | 0 | 0.0 | 12 | 18.5 | 12 | 6.7 | <0.001 |
| Distance form hospital | 1 | 0.9 | 14 | 21.5 | 15 | 8.3 | <0.001 |
| Maternal education | 0 | 0.0 | 8 | 12.3 | 8 | 4.4 | 0.001 |
| Median 7-item socio-economic scores (IQR) | 1 | 0 - 2 | 4 | 1.75 - 4.25 | 2 | 1 - 4 | <0.001 |
| **Types of physical exams: Performed and documented** |  |  |  |  |  |  |  |
| **Physical measures** |  |  |  |  |  |  |  |
| Weight (kg) | 104 | 90.4 | 55 | 84.6 | 159 | 88.3 | 0.354 |
| Temperature | 92 | 80.0 | 59 | 90.8 | 151 | 83.9 | 0.094 |
| Respiratory rate | 42 | 36.5 | 1 | 1.5 | 43 | 23.9 | <0.001 |
| Heart rate | 40 | 34.8 | 2 | 3.1 | 42 | 23.3 | <0.001 |
| Pulse oximetry | 39 | 33.9 | 0 | 0.0 | 39 | 21.7 | <0.001 |
| Mid-upper arm circumference | 32 | 27.8 | 29 | 44.6 | 61 | 33.9 | 0.022 |
| Length or height (cm) | 18 | 15.7 | 25 | 38.5 | 41 | 22.8 | 0.001 |
| Blood pressure | 1 | 0.9 | 11 | 16.9 | 12 | 6.7 | <0.001 |
| Median 8-item physical score | 3 | 2 - 4 | 3 | 2 - 4 | 3 | 2 - 4 | 0.201 |
| **Clinician observed measures** |  |  |  |  |  |  |  |
| Check for severe pallor | 85 | 73.9 | 1 | 1.5 | 86 | 47.8 | <0.001 |
| Check for jaundice | 78 | 67.8 | 21 | 32.3 | 99 | 55.0 | <0.001 |
| Central cyanosis | 60 | 52.2 | 0 | 0.0 | 60 | 33.3 | <0.001 |
| Check for convulsions, lethargy, consciousness | 59 | 51.3 | 2 | 3.1 | 61 | 33.9 | <0.001 |
| Check for generalized weakness | 58 | 50.4 | 29 | 44.6 | 87 | 48.3 | 0.552 |
| Auscultation of chest, wheezing | 55 | 47.8 | 0 | 0.0 | 55 | 30.6 | <0.001 |
| Check for respiratory distress | 53 | 46.1 | 1 | 1.5 | 54 | 30.0 | <0.001 |
| Check for abdominal distension | 50 | 43.5 | 6 | 9.2 | 56 | 31.1 | <0.001 |
| Check for abdominal mass | 48 | 41.7 | 3 | 4.6 | 51 | 28.3 | <0.001 |
| Check for sunken eyes | 40 | 34.8 | 15 | 23.1 | 55 | 30.6 | 0.142 |
| Skin pinch | 30 | 26.1 | 5 | 7.7 | 35 | 19.4 | 0.003 |
| Apex beat displaced/trachea | 28 | 24.3 | 0 | 0.0 | 28 | 15.6 | <0.001 |
| Capillary refill time | 21 | 18.3 | 5 | 7.7 | 26 | 14.4 | 0.086 |
| Check for cool peripheries | 18 | 15.7 | 1 | 1.5 | 19 | 10.6 | 0.003 |
| Median 14-item observed score | 5 | 2 - 10 | 1 | 0 - 3 | 3 | 0 - 7 | <0.001 |
| **In-patient consultation topics: Discussed and documented** |  |  |  |  |  |  |  |
| Type of patient illness | 44 | 38.3 | 49 | 75.4 | 93 | 51.7 | <0.001 |
| Prescriptions | 21 | 18.3 | 47 | 72.3 | 68 | 37.8 | <0.001 |
| Patient care management | 21 | 18.3 | 47 | 72.3 | 68 | 37.8 | <0.001 |
| Estimated length of stay | 10 | 8.7 | 22 | 33.8 | 32 | 17.8 | <0.001 |
| Challenges they may face: cost of prescriptions | 5 | 4.3 | 0 | 0.0 | 5 | 2.8 | 0.161 |
| Challenges they may face during treatment: Cost of food | 4 | 3.5 | 0 | 0.0 | 4 | 2.2 | 0.298 |
| Median 6-item in-patient consultation score | 0 | 0 - 2 | 3 | 2 - 4 | 1 | 0 - 3 | <0.001 |
| Missing data impacted denominator. Denominator as follows: : ^a^n=113; ^b^n=64; ^c^n=177; ^d^n=114; ^e^n=179; | | | | | | | |

# Table D. Caregiver and health worker satisfaction with discharge care.

|  | **Hospitals (n=115)** | | **Health Centres (n=65)** | | **p-value** | **PNFP (n=40)** | | **Public (n=140)** | | **p-value** | **All (n=180)** | |
| --- | --- | --- | --- | --- | --- | --- | --- | --- | --- | --- | --- | --- |
|  | **n** | **%** | **n** | **%** |  | **n** | **%** | **n** | **%** |  | **n** | **%** |
| **Caregiver Satisfaction** | | | | | | | | | | | | |
| Health workers spent enough time preparing caregiver for discharge | 58 | 50.4 | 47 | 72.3 | 0.004 | 24 | 60.0 | 81 | 57.9 | 0.808 | 105 | 58.3 |
| Caregiver was involved in their child's discharge as much as they wanted to be | 56 | 48.7 | 41 | 63.1 | 0.063 | 18 | 45.0 | 79 | 56.4 | 0.201 | 97 | 53.9 |
| Discharge time was convenient | 95 | 82.6 | 52 | 80.0 | 0.664 | 34 | 85.0 | 113 | 80.7 | 0.537 | 147 | 81.7 |
| Caregiver felt their child was fit to be discharged | 105 | 91.3 | 53 | 81.5 | 0.055 | 34 | 85.0 | 124 | 88.6 | 0.543 | 158 | 87.8 |
| Caregiver received a referral for follow-up care | 38 | 33.0 | 21 | 32.3 | 0.92 | 16 | 40.0 | 43 | 30.7 | 0.27 | 59 | 32.8 |
| Among those who received a referral, caregiver was referred to their preferred facility | 34 | 89.5 | 21 | 100.0 | 0.124 | 16 | 100.0 | 39 | 90.7 | 0.206 | 55 | 93.2 |
| **Health Worker Satisfaction** | | | | | | | | | | | | |
| **Overall, health worker is pleased with facility's discharge process:** | | | | | | | | | | | | |
| Always | 5 | 4.3 | 8 | 12.3 | 0.010 | 3 | 7.5 | 10 | 7.1 | 0.310 | 13 | 7.2 |
| Often | 37 | 32.2 | 31 | 47.7 |  | 12 | 30.0 | 56 | 40.0 |  | 68 | 37.8 |
| Sometimes | 60 | 52.2 | 19 | 29.2 |  | 23 | 57.5 | 56 | 40.0 |  | 79 | 43.9 |
| Rarely | 13 | 11.3 | 6 | 9.2 |  | 2 | 5.0 | 17 | 12.1 |  | 19 | 10.6 |
| Never | 0 | 0.0 | 1 | 1.5 |  | 0 | 0.0 | 1 | 0.7 |  | 1 | 0.6 |
| **Perceived peer satisfaction:** | | | | | | | | | | | | |
| Number of health workers who perceived the majority of their colleagues were satisfied with the facility's discharge process | 51^a^ | 44.7 | 42 | 64.6 | 0.010 | 19^b^ | 48.7 | 74 | 52.9 | 0.647 | 93 | 51.7 |
| **The number of staff available to discharge children is:** | | | | | | | | | | | | |
| Plenty | 7 | 6.1 | 6 | 9.2 | 0.008 | 5 | 12.5 | 8 | 5.7 | 0.031 | 13 | 7.2 |
| Satisfactory | 31 | 27.0 | 31 | 47.7 |  | 17 | 42.5 | 45 | 32.1 |  | 62 | 34.4 |
| Occasionally inadequate | 42 | 36.5 | 20 | 30.8 |  | 15 | 37.5 | 47 | 33.6 |  | 62 | 34.4 |
| Usually Inadequate | 35 | 30.4 | 8 | 12.3 |  | 3 | 7.5 | 40 | 28.6 |  | 43 | 23.9 |
| **The number of staff available to discharge children on the weekends is:** | | | | | | | | | | | | |
| Plenty | 1 | 0.9 | 9 | 13.8 | <0.001 | 2 | 5.0 | 8 | 5.7 | 0.721 | 10 | 5.6 |
| Satisfactory | 27 | 23.5 | 33 | 50.8 |  | 16 | 40.0 | 44 | 31.4 |  | 60 | 33.3 |
| Occasionally inadequate | 43 | 37.4 | 16 | 24.6 |  | 13 | 32.5 | 46 | 32.9 |  | 59 | 32.8 |
| Usually Inadequate | 44 | 38.3 | 7 | 10.8 |  | 9 | 22.5 | 42 | 30.0 |  | 51 | 28.3 |
| **The availability of support or supervision when discharging a child is:** | | | | | | | | | | | | |
| Plenty | 17 | 14.8 | 18 | 27.7 | 0.040 | 8 | 20.0 | 27 | 19.3 | 0.592 | 35 | 19.4 |
| Satisfactory | 69 | 60.0 | 28 | 43.1 |  | 24 | 60.0 | 73 | 52.1 |  | 97 | 53.9 |
| Occasionally inadequate | 20 | 17.4 | 9 | 13.8 |  | 6 | 15.0 | 23 | 16.4 |  | 29 | 16.1 |
| Usually Inadequate | 9 | 7.8 | 10 | 15.4 |  | 2 | 5.0 | 17 | 12.1 |  | 19 | 10.6 |
| **Health worker rating of the caregiver post-discharge education provided at their facility:** | | | | | | | | | | | | |
| Good | 27 | 23.5 | 26 | 40.0 | 0.088 | 13 | 32.5 | 40 | 28.6 | 0.649 | 53 | 29.4 |
| Satisfactory | 26 | 22.6 | 15 | 23.1 |  | 8 | 20.0 | 33 | 23.6 |  | 41 | 22.8 |
| Needs improvement | 49 | 42.6 | 20 | 30.8 |  | 17 | 42.5 | 52 | 37.1 |  | 69 | 38.3 |
| Inadequate | 13 | 11.3 | 4 | 6.2 |  | 2 | 5.0 | 15 | 10.7 |  | 17 | 9.4 |
| Missing data: ^a^n=114; ^b^n=39 | | | | | | | | | | | | |

# Fig A. Availability of facility resources by service delivery level and ownership type, n=36 facilities


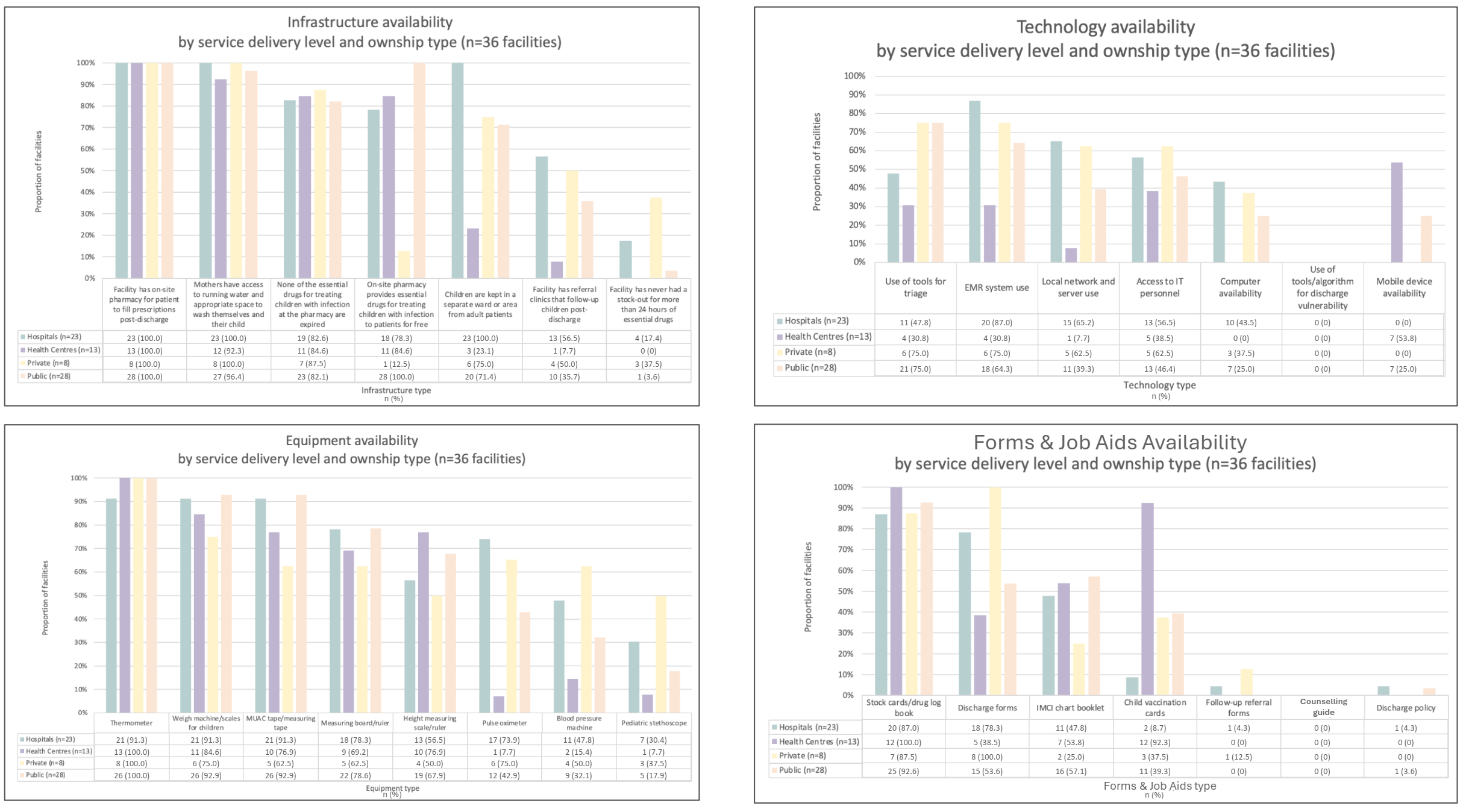


# Fig B1-5: Observed health worker care practices at admission, n=180 observations

## Fig B1: Medical history taking: Asked and documented


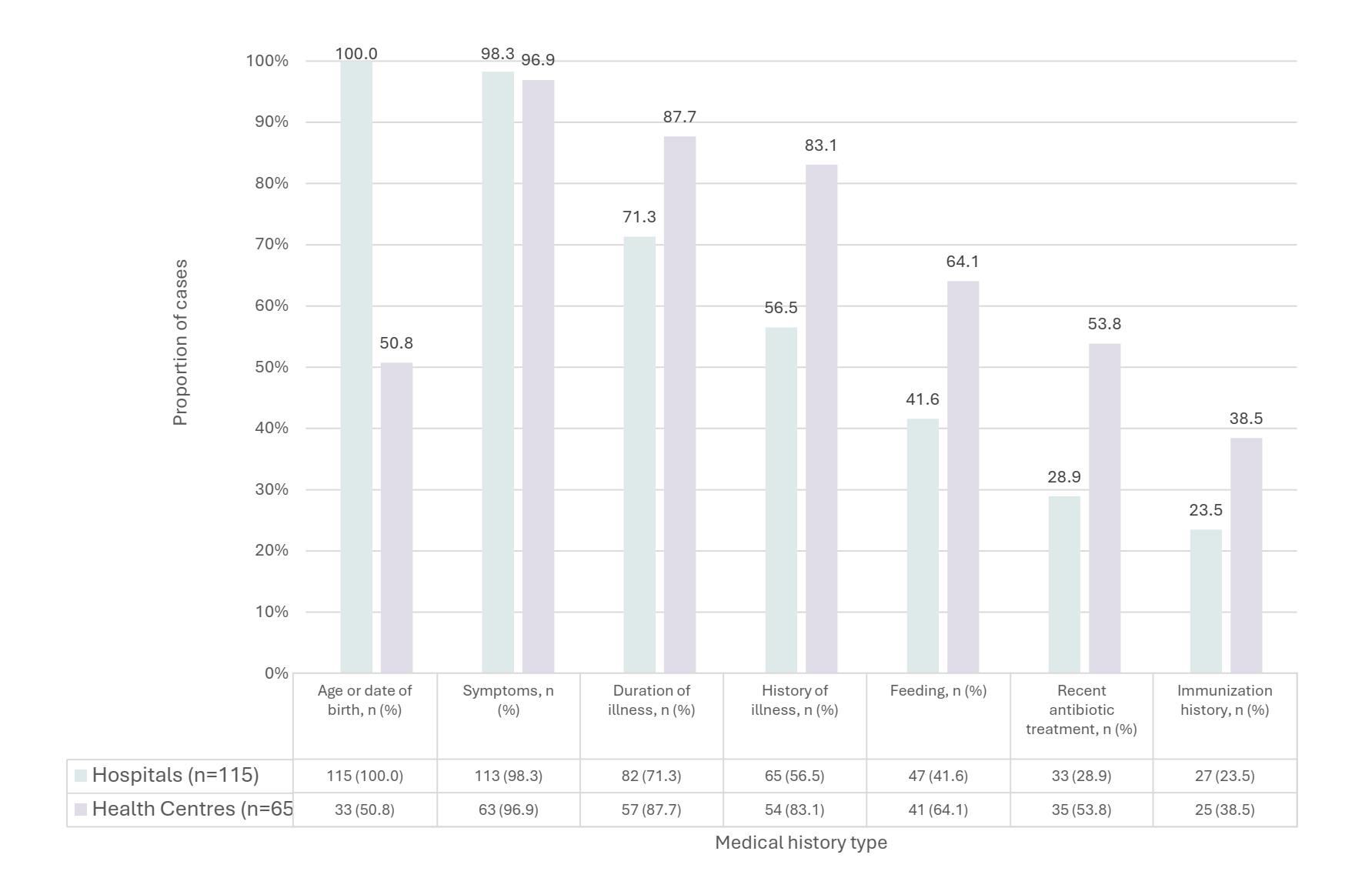


## Fig B2: Socio-economic history taking: Asked and documented


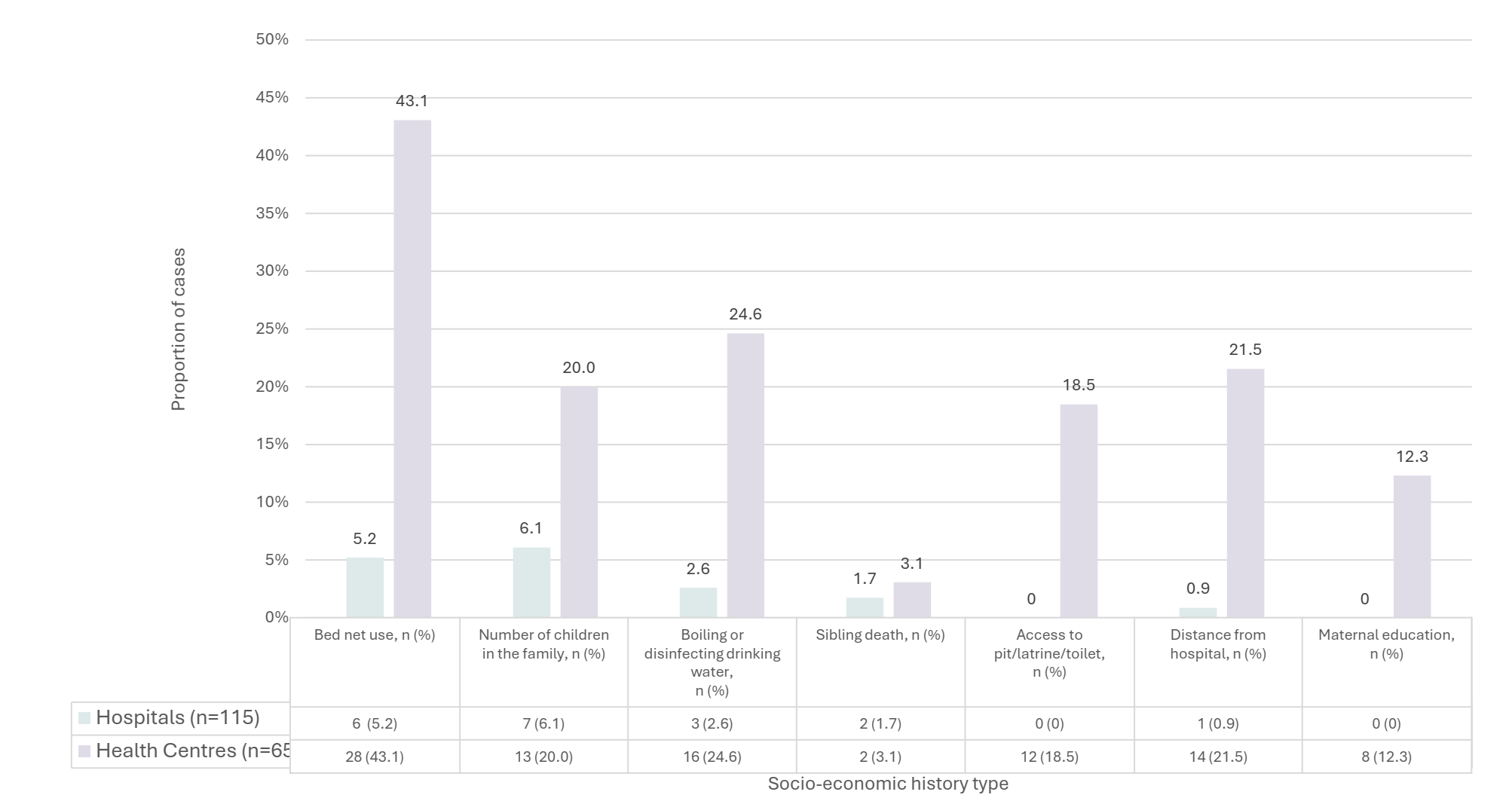


## Fig B3: Physical measures: Performed and documented


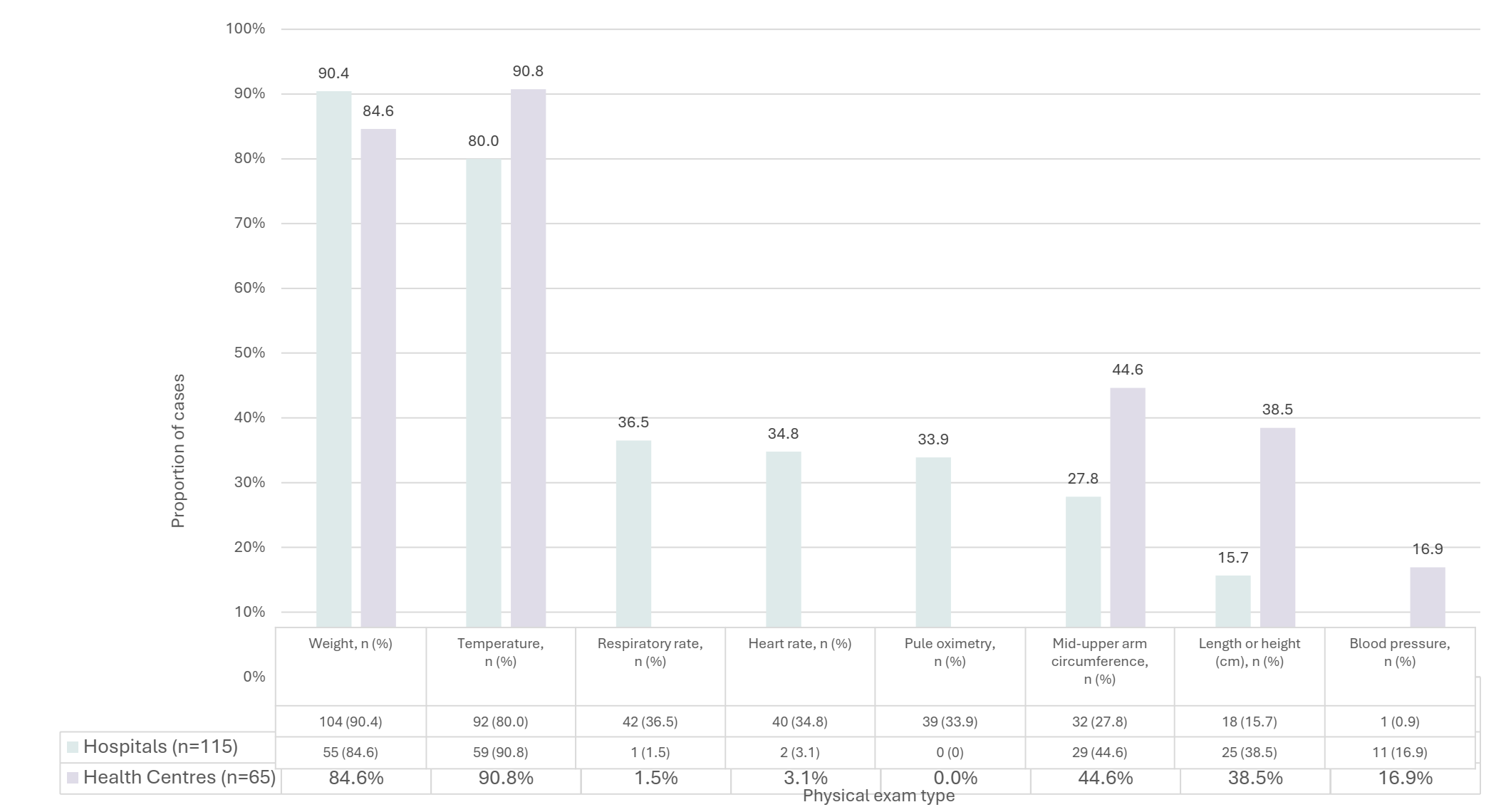


## Fig B4: Clinician observed measures: Performed and documented


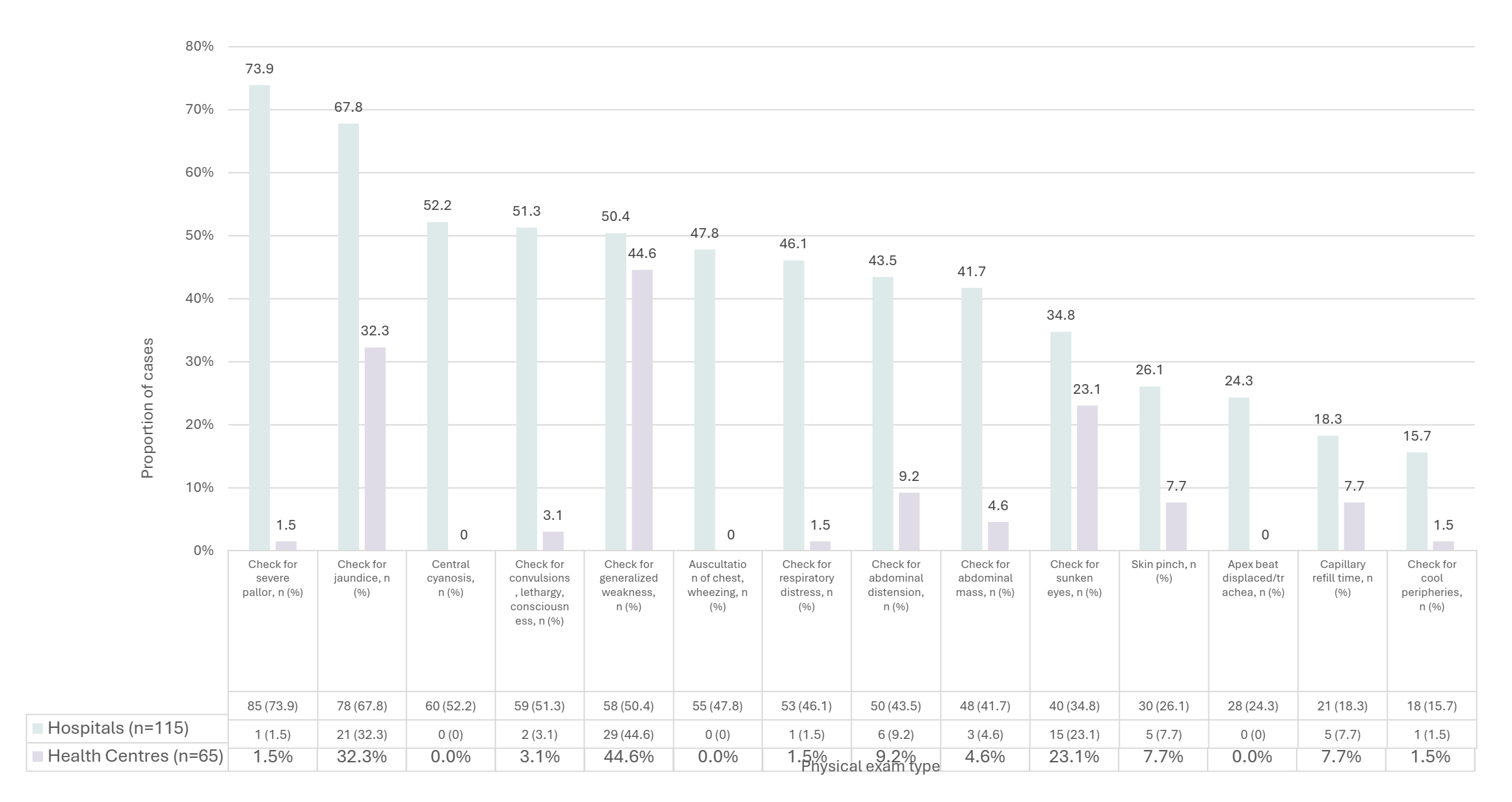


## Fig B5: Admission consultation topics discussed with caregiver


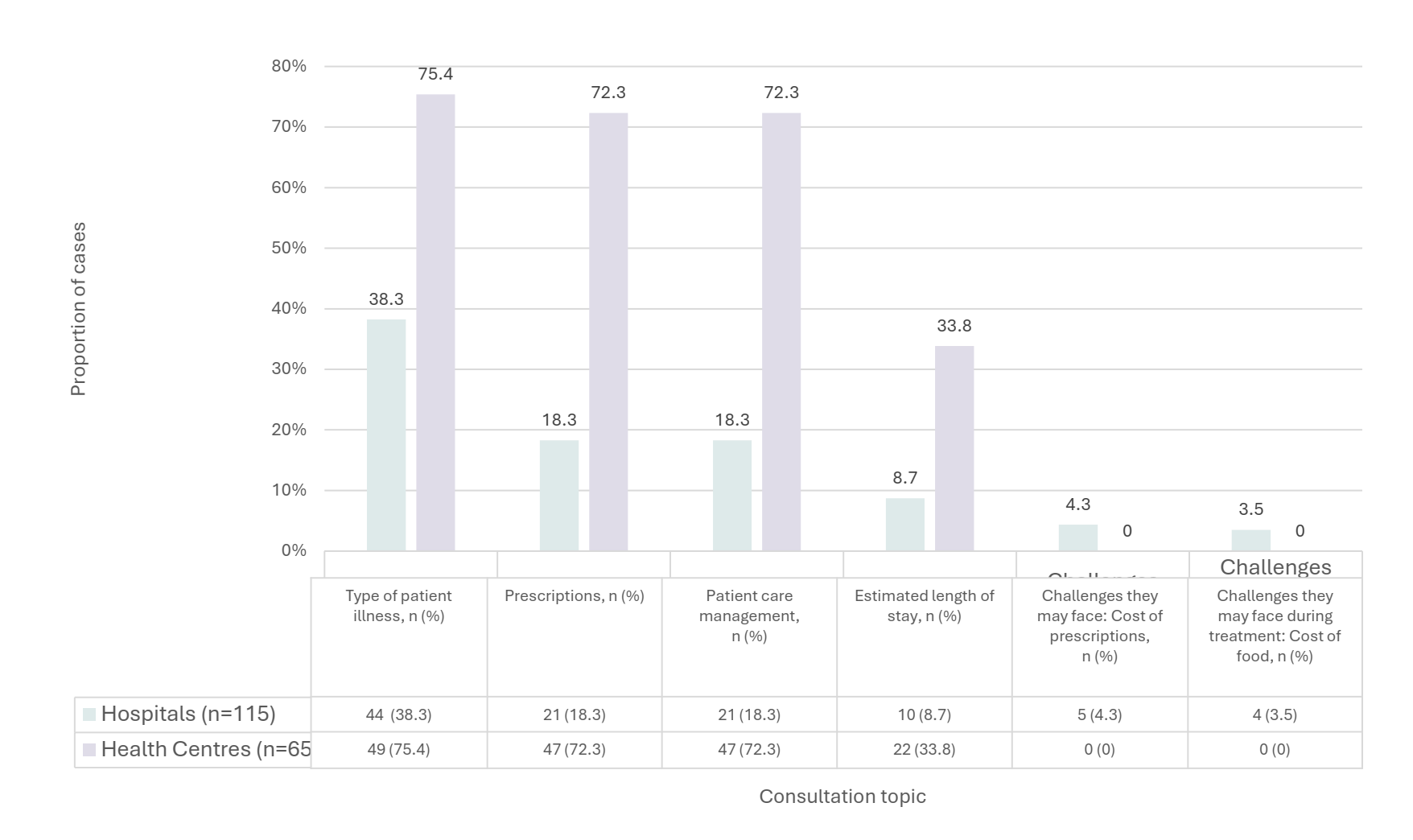

Supplement: S1 Text — (DOCX) [file pgph.0003559.s001.docx]
