# Supplementary material for: Discharge policies and care practices for children with suspected sepsis: A health facility scan at a nationally representative sample of hospitals and health centres in Uganda
Source: PLOS Glob Public Health. 2025 Aug 22;5(8):e0003559. doi: 10.1371/journal.pgph.0003559 (PMC12373204; doi:10.1371/journal.pgph.0003559)
Supplement: Attachment — Submitted filename: FScan_Response_to_reviewers_FINAL.docx [file pgph.0003559.s003.docx]

**Date: January 15**

Dear Editor and Reviewers,

We are pleased to submit our revised manuscript, titled “Discharge policies and care practices for children with suspected sepsis: A health facility scan at a nationally representative sample of hospitals and health centres in Uganda,” for consideration for publication in PLOS Global Health.

We extend our sincere thanks to the Editorial Board and reviewers for their insightful comments and valuable suggestions. Enclosed, you will find our responses and requested revisions, which we believe have significantly enhanced the quality and clarity of the manuscript. We appreciate your time and look forward to your deliberation.

Thank you for your consideration.

Sincerely,

Dr. Matthew O. Wiens, PhD; PharmD

Assistant Professor

Department of Anesthesiology, Pharmacology & Therapeutics | Institute for Global Health

The University of British Columbia | BC Children's Hospital and BC Women’s Hospital + Health Centre

938 W 28th Ave | Vancouver BC | V5Z 4H4 Canada

Phone: 604 997 7753; Email: mowiens@outlook.com

**RESPONSE TO REVIEWERS**

**Manuscript reference number:** PLOS GLOBAL PUBLIC HEALTH PGPH-D-24-01608

**Title:** Discharge policies and care practices for children with suspected sepsis: A health facility scan at a nationally representative sample of hospitals and health centres in Uganda

**Journal Requirements:**

**Comment 1.** Thank you for providing the requested questionnaire for global inclusivity. However, we note that the questionnaire has been submitted as an ""other"" file type. Kindly resubmit the global inclusivity questionnaire as Supporting file.

**Response:** Thank you for your guidance. We apologize for the oversight and have now resubmitted the global inclusivity questionnaire as a Supporting file as requested.

**Comment 2.** In the online submission form, you indicated that "Access to these data will be granted on a case-by-case basis following approval from the authors and the Data Governance Committees." All PLOS journals now require all data underlying the findings described in their manuscript to be freely available to other researchers, either 
1. In a public repository, 
2. Within the manuscript itself, or 
3. Uploaded as supplementary information.

This policy applies to all data except where public deposition would breach compliance with the protocol approved by your research ethics board. If your data cannot be made publicly available for ethical or legal reasons (e.g., public availability would compromise patient privacy), please explain your reasons by return email and your exemption request will be escalated to the editor for approval. Your exemption request will be handled independently and will not hold up the peer review process, but will need to be resolved should your manuscript be accepted for publication. One of the Editorial team will then be in touch if there are any issues.

**Response:** Thank you for highlighting this important aspect. All study materials, including the protocol, data collection tools, data dictionary, and metadata, are openly accessible through the [Pediatric Sepsis Data CoLaboratory’s (Sepsis CoLab)](https://borealisdata.ca/dataset.xhtml?persistentId=doi:10.5683/SP3/IDLGNN) Dataverse on Borealis, the Canadian Dataverse Repository. We obtained ethical approval to deposit de-identified data on a Sepsis CoLab’s Dataverse repository with moderated access due to the sensitive nature of clinical data and the potential for re-identification. Data access requests will be carefully reviewed and granted on a case-by-case basis, requiring approval from both the authors and the Data Governance Committees.

**Additional Editor Comments (if provided):** This research represents an important and underpublished topic that sheds light onto discharge practices in Uganda. The manuscript needs significant edits particularly to strengthen the methodology and conclusions. **Response:** Thank you for recognizing the importance of our research and for your thoughtful feedback. We appreciate the reviewers' detailed comments and have made substantial revisions to strengthen the methodology, clarify the conclusions, and improve overall clarity throughout the manuscript. We believe these revisions have addressed the reviewers' suggestions, and we look forward to your assessment of the revised submission.

**REVIEWER #1**

**Comment:** This paper assesses the readiness of health facilities in Uganda to manage pediatric discharge care for children under 5 years or age, focusing 36 representative health facilities. The study employed five distinct surveys to assess facility resources, technological preparedness, quality and safety during severe illness/infection, patient-caregiver perceptions, and health worker perceptions of care. The findings reveal discrepancies in discharge counseling practices between health centers and hospitals. Specifically, health centers had more robust discharge counseling and higher caregiver satisfaction despite lower infrastructure domain scores, whereas hospitals provided more post-discharge risk assessments and scheduled follow up more frequently. These results identify critical areas for quality improvement and can offer valuable guidance for shaping national level pediatric discharge policy in Uganda. The study has an impressive scope and is well-designed, thorough, and well-written, making it a strong candidate for publication.

**Response:** Thank you for your positive and encouraging feedback. We are pleased that you found the scope, design, and findings of our study valuable. We have carefully addressed your insightful feedback in our manuscript and provided responses to each of your comments below.  **Recommendations:
Intro:
Comment 1:** Clarify that the study specifically targets pediatric discharge care for children under 5 years old in sections such as the abstract and introduction to make it clear for the readers.

**Response:** We apologize for this oversight. The abstract (Page 2, Line 27) and Intro (Page 5, Line 75) have ben updated to state that the study specifically targets pediatric discharge care for children under 5 yeas of age.

**Comment 2:** Page 4, line 70: explicitly state how the study aligns with or utilizes WHO guidelines for effective hospital discharge, as this connection is currently unclear.

**Response:** Thank you for this feedback. Page 4, line 71 has been updated to make this connection more clear by stating: “This study aligns with WHO guidelines by evaluating how well health facilities in Uganda implement these core elements, providing a health systems-based assessment of existing resources and practices in pediatric discharge.”

**Methods:
Comment 3**: Define the primary outcome more clearly. The term “discharge readiness” is unclear without a defined metric or validated scales. Consider focusing on outcomes such as resource availability and the observation of discharge care practices. Ensure these primary outcomes are referenced in the abstract and conclusion/discussion for clarity.

**Response:** We agree that the term discharge readiness is perhaps not the most appropriate terms for reasons that you have described. Discharge readiness is not meant to convey a patient’s suitability for discharge, but rather a facilities readiness to provide optimal discharge care. Like the scan in general, this metric is not validated, and have mentioned this in our limitations section. This limitation also is present within the subset of features we used to define our primary outcome. On page 8, line 159, we have changed the primary outcome to the following:

“Primary outcomes for this study included facility readiness for discharge, defined as resource availability and observation of four key discharge care practices: i) post-discharge risk assessment; ii) referrals; iii) provision of take-home materials; iv) post-discharge counselling.”

**Comment 4:** Please include the modified questions from the Sepsis CoLab Environmental Scans in the supplemental files, as these modifications pertain to discharge care. Currently, they are under moderated access.

**Response:** Thank you for your suggestion. The data collection tools used in this study are available through the Sepsis CoLab’s Dataverse repository, which is accessible to all CoLab members. While they are currently under moderated access, anyone can obtain access by completing a brief membership application. These tools have been contributed to the Sepsis CoLab in appreciation for their use in this study as well as to foster collaboration. Additionally, Dataverse provides version control, ensuring transparency and enabling us to cite the tools in future publications. This approach allows us to continue refining the discharge module while maintaining a centralized, up-to-date repository.

**Comment 5:** If an assessment of pediatric vital signs at discharge was conducted (ie obtained vs not obtained, normal vs abnormal), please include this information. If not, consider discussing the absence of this data in the discussion section.

**Response:** Thank you for this valuable suggestion. We have updated Page 14, lines 270-272, and Table 2 to include information about whether health workers conducted clinical assessments on the day of discharge, which was recorded as a binary yes/no variable. While we assessed whether an assessment was performed, the data did not capture the assessment results or specify which assessments were conducted. We have now acknowledged this as a limitation in the discussion (Page 23, lines 429-431) and have provided feedback to the Sepsis CoLab to consider enhancing the Observational Scan tool to capture these additional details in future studies.

**Results:
Comment 6:** Infrastructure domain scores are first mentioned on Page 11, line 208. Please provide a detailed explanation in the methods section regarding how these scores were derived and what they encompass.

**Response:** Please see the Statistical analysis section on Page 9, lines 162-177 which provides a detailed explanation about domain scores. S2 Table and S1 Figure also provide a breakdown of all items included in each of the 4 domains and provide statistics on each item assessed within each domain, and the final domain index score (median, IQR).

**Comment 7:** Page 10, Lines 195-196: clarify the discrepancy in patient numbers (n=180 discharged patients vs. n = 145 in the following line). Consider revising the total N to 145 if appropriate.

**Response:** Apologies for the confusion. The discrepancy is due to missing data. Indeed, we observed 180 pediatric patients as presented in the opening statement. However, some variables have missing data. We attempted to provide clarity around this by providing both the numerator and denominator when reporting outcomes with missingness. E.g. “Health workers were observed discharging 180 pediatric patients who had been admitted with suspected sepsis. Among these patients, the majority (n=142/145; 97.9%) were routinely discharged.” All figures and tables report missingness as needed.

**Comment 8:** Address if any enrolled facilities were omitted from the dataset for observation of discharge care practices (as n = 145) and discuss how this might affect the study’s findings.

**Response:** Thank you for this comment. No facilities were omitted from the dataset for observation of discharge care practices. As mentioned above, some variables included in the observation assessment had missing data; therefore, we reported missing data, when necessary, in the tables and figures. Other observation variables, such as all 5 key discharge care practices (Table 2), had no missingness (n=180). We updated the statement about missing data in the Statistical analysis section (Page 9, line 176-177), to state: “Missing data were removed from the analysis and reported throughout the manuscript as numerators over denominators to clarify the variables affected by missing data.”

**Comment 9:** Page 10, Lines 197 (or Figure 1): indicate the nature of the diagnoses for the remaining 37.9% of patients not treated for malaria or pneumonia, as this represents the second-largest category.

**Response:** Thank you. The “other or multiple infections” category includes patients diagnosed with infections other than malaria or pneumonia, as well as those with multiple infections, which may include malaria and/or pneumonia and/or other infections (e.g. abscess). Due to the infrequency of these other diagnoses and the fact that the manuscript is already exceptionally long, we chose to group them under “other or multiple infections” to maintain clarity and conciseness. We have revised the manuscript to clarify this point (page 11, line 199), stating: “All other diagnoses (n=57/145; n=37.9%) were reported as other (e.g., septicemia) or multiple infections (e.g., malaria and hepatitis B) but were not frequent enough to report independently.”

**Other comments:** A thorough copy-editing is recommended to correct minor spelling and grammatical errors. For instance, line 52 should use “sub-Saharan” rather than “Saharan”, line 204: “enrolment” is misspelled, line 271-273 “whereas…compared to hospital health workers.” should be a complete sentence, Table 2 should list “n” as 65 under “discharge counseling topics, hospitals”. Additionally, consider improving the quality of the figures, which appear slightly grainy.

Overall, this manuscript provides significant insights into pediatric discharge readiness in low and middle income countries and has the potential to make a substantial contribution to the literature.

**Response:** Thank you for your helpful comments. We have carefully addressed all of your suggestions while conducting a thorough copy edit of the manuscript. We corrected the spelling and grammatical errors you identified, including changing “Saharan” to “sub-Saharan” on line 52, correcting the spelling of “enrolment” on line 204, and ensuring that the statement on lines 271-273 is now a complete sentence. Additionally, we updated Table 2 to accurately list “n” as 65 under “discharge counseling topics, hospitals.” We also took your feedback into account and improved the quality of the figures to enhance their clarity. We appreciate your recognition of the manuscript's potential contribution to the literature on pediatric discharge readiness in low- and middle-income countries.

**REVIEWER #2:** 

**Comment 1:** It’s overall unclear to me what the big take-home messages are, from this analysis, that are actionable either at a policy level or in the next phase of research into this problem.

**Response:** Thank you for your feedback. We have made several revisions throughout the discussion section and strengthened the conclusion (new text in bold) to more clearly state the take-home messages.

“The findings from this facility scan have pinpointed key areas for quality improvement in both resource availability and effective provision of discharge care. Despite having fewer resources, health centres demonstrated better discharge care practices and higher satisfaction than hospitals, highlighting the need to investigate factors contributing to these successful practices and exploring their applicability in hospitals. Targeted interventions, such as **standardized job aids and discharge policies that encompass objective risk assessments, discharge counselling, take-home materials, and referrals, as well as improved health worker training and job aid availability,** are crucial to address the challenges identified. These insights can serve as a roadmap for shaping a national-level pediatric discharge policy in Uganda which **may ultimately improve post-discharge outcomes.”**

**Comment 2:** Would recommend having someone do a general edit for writing clarity and appropriate use of abbreviations (most notably “n”). The paper needs a close grammar check; some sentences are awkwardly worded and subject-verb agreement is sometimes off. Also would change passive voice to active voice throughout; the passive voice makes it much more difficult to follow your methods and results.

**Response:** Thank you for your feedback. We have done a thorough copy edit of the manuscript, reduced passive voice, and ensured consistent and appropriate use of "n." We trust these edits improve the writing clarity and ease of following the methods and results.

**Comment 3:** Clarify criteria for any purposive sampling, and qualitative methods generally.

**Response:** Thank you for your feedback. With regards to purposive sampling, we have attempted to describe in detail our approach to facility selection. Ultimately, our experience and organizational network facilitated our choice of facilities, but this was done with a specific motivation of achieving as much of a representative sample as possible. As described in the methods, “We selected 36 health facilities to comprise a sample that was nationally representative of the Ugandan health system and the varying socio-demographics of the local population. A district-level sampling frame was used to select lower-level health facilities, including 3 hospitals and 13 health centres from 2 districts in Northern and Western Uganda whereas a national-level sampling frame was used to select 20 hospitals from 20 districts across Uganda (Table 1). Study sites have both public and private not-for-profit (PNFP) ownership as well as rural and urban geographical representation.” We hope this better clarifies the context of our sampling

As suggested by several reviewers, we have removed the qualitative components from this manuscript and aim to report these findings in a separate manuscript.

**Comment 4:** It’s unclear to me how the authors have determined that this is a nationally representative sample; this assertion needs further explanation, as they have excluded one segment of the health facilities and have not represented all regions of the country.

**Response:** We apologize for the lack of clarity. We have updated page 5 to provide more explanation, stating “We used both district- and national-level sampling frames to capture a representative sample of the Ugandan health system’s diverse structure and to ensure balanced comparisons across service delivery levels and ownership types. For lower-level health facilities, we selected 3 hospitals and 13 health centres from two districts in Northern and Western Uganda, chosen for their demographic similarities, including a high proportion of children under five, comparable healthcare access, and varied healthcare infrastructure (Table 1). This district-level sampling allowed for a focused examination of local healthcare contexts with shared population characteristics. In contrast, to capture the variability of hospitals across Uganda, we used a national-level sampling frame to select 20 hospitals from 20 districts. This broader sampling approach ensured that our hospital sample reflected a wide range of geographical and operational contexts across the country. Study sites include both public and private not-for-profit (PNFP) ownership types, representing both rural and urban geographical settings. Facilities were eligible for inclusion if they provided pediatric in-patient care and had no prior involvement in the Smart Discharges research program [16]. Private-for-profit health facilities were excluded.”

**Comment 5:** Decrease references to supplementary materials; consider separating into multiple manuscripts if there are so many important results so as to be difficult to pare down to a reasonable number of tables and figures for inclusion in the text.

**Response:** Thank you for your feedback. We have removed the qualitative components from this manuscript and aim to report these findings in a separate manuscript, as suggested by several reviewers.

**Comment 6:** Avoid reporting trends in data that were not significant, especially with this much data to parse.

**Response:** Thank you for your feedback. We have reviewed the results section and removed statements reporting trends in data that were not significant.

**Comment 7:** Qualitative methods and themes are inadequately described. What were your qualitative questions?

**Response:** Thank you for your feedback. We have removed the qualitative components from this manuscript and aim to report these findings in a separate manuscript, as suggested by several reviewers.

**Comment 8:** Please address whether there are any specific conclusions about particular policies that might impact post-discharge mortality (e.g., follow-up appointments not being routine), that aren’t currently widely in place? Are there any SPECIFIC interventions that emerge from your data, that Uganda could trial?

**Response:** Thank you for your feedback. Similar to our response to Comment 1, we have made several revisions throughout the discussion section and strengthened the conclusion (new text in bold) to provide specific conclusions about policies that could impact post-discharge mortality.

“The findings from this facility scan have pinpointed key areas for quality improvement in both resource availability and effective provision of discharge care. Despite having fewer resources, health centres demonstrated better discharge care practices and higher satisfaction than hospitals, highlighting the need to investigate factors contributing to these successful practices and exploring their applicability in hospitals. Targeted interventions, such as **standardized job aids and discharge policies that encompass objective risk assessments, discharge counselling, take-home materials, and referrals, as well as improved health worker training and job aid availability,** are crucial to address the challenges identified. These insights can serve as a roadmap for shaping a national-level pediatric discharge policy in Uganda which **may ultimately improve post-discharge outcomes.”**

**Minor comments and revisions:

Abstract:
Comment 9:** What is a “facility scan?” Did you use a validated instrument?

**Response:** The Facility Scan is a 5-survey assessment tool to support facilities in in identifying quality improvement priorities in treating children with sepsis. It was informed by the Pediatric Sepsis Data CoLab’s Environmental Scan tools. Although these tools were not validated, they were developed by pediatric sepsis experts working in LMICs (Fung et al. 2022) and have been piloted in 6 African hospitals (Hwang et al 2024). We have included additional information (in bold) in the Data Collection Tool section where we describe the facility scan.

“All study sites underwent a 5-survey Facility Scan informed by the Pediatric Sepsis Data CoLaboratory’s (Sepsis CoLab) Environmental Scan which is used to support health facilities in identifying quality improvement priorities in treating children with sepsis (Fung et al 2022). These five surveys are comprised of i) a Scan to assess facility resources; ii) a Scan to assess a facility’s level of technological preparedness; iii) a Scan to assess the quality and safety of care through observation of a health worker in suspected or confirmed cases of severe illness/infection; iv) a Caregiver Satisfaction Questionnaire to assess patient-caregiver perceptions of the quality of care received at the facility; and v) a Health Worker Satisfaction Questionnaire to assess health worker perceptions of the quality of care provided a the facility (S1 Table). **The Environmental Scan was developed by 12 emergency medicine and critical illness experts in LMICs (Fung et al, 2022) and piloted in six African hospitals and demonstrated feasibility for assessing priority areas to enhance quality of care for critically ill children in low-resource settings (Hwang et al. 2024).**

Each of the five Sepsis CoLab Environmental Scans were enhanced to address specific areas (including discharge planning, post-discharge counselling, provision of take-home material, and follow-up referrals) relevant to discharge care through consultations with the local study team members which included pediatric acute and critical care investigators and health workers. We also omitted the non-discharge relevant aspects of the original scans. The discharge modules designed for this Facility Scan have since been incorporated in the Sepsis CoLab’s Environmental Scan, which is publicly available via Borealis, the Canadian Dataverse Repository.”

- Fung JST, Hwang B, Dunsmuir D, et al. A 2-Phase Survey to Assess a Facility’s Readiness for Pediatric Essential Emergency and Critical Care in Resource-Limited Settings: A Literature Review and Survey Development. Pediatr Emerg Care. 2022;38: 532–539. doi:10.1097/PEC.0000000000002826
- Hwang B, Trawin J, Dzelamunyuy S, Et al. Assessment of Facility Readiness for Pediatric Emergency and Critical Care Utilizing a 2-Phase Survey Conducted in Six Hospitals in Uganda and Cameroon: A Quality Improvement Study. Pediatric Emergency Care ():10.1097/PEC.0000000000003276, November 6, 2024.. DOI: 10.1097/PEC.0000000000003276

**Comment 10:** Were private for-profit facilities assessed?

**Response:** Private-for-profit facilities were excluded. Please see Page 5, Line 93 which states “Facilities were eligible for inclusion if they provided pediatric in-patient care and had no prior involvement in the Smart Discharges research program. Private-for-profit health facilities were excluded.”  **Introduction:
Comment 11:** Line 59 – typo: “require,” not “requires”

**Response:** Thank you for identifying this error. We have corrected the typo to state “require”.

**Comment 12**: You note that little work has been done in LMICs on discharge practices, but one of the only papers you cite that supports the assertation that discharge his a “complex and error-prone process” is actually from Uganda. Is there other literature on the discharge process from HICs that you could cite, or do we just not know a lot about what makes a safe discharge in general?

**Response:** Thank you for this insightful comment. The Uganda study we cited was conducted by our team to better understand discharge practices in the regions where we work. While limited evidence on discharge processes exists from high-income countries (HICs), we chose not to include these references due to the considerable differences in available resources, which limits their applicability to low- and middle-income countries (LMICs). Instead, we focused on the WHO Guidelines for the Management of Common Childhood Illnesses, which outlines key components of effective discharge care relevant to LMICs (Lines 70-73). Additionally, we have referenced a recently published systematic review (Rossi et al. 2024) on safe pediatric discharges. This review highlights that a comprehensive definition of “safe discharge” is still lacking, and it notes that essential elements for safe pediatric discharge remain unclear and are often not applicable across conditions. We believe this further supports our approach of using the WHO Guidelines as a framework for safe discharge practices in resource-limited settings.

- Rossi S, Hayter M, Zuco A, Tappino F, Tirone R, Scelsi S. Essential elements nurses have to address to promote a safe discharge in paediatrics: A systematic review and narrative synthesis. Nurs. open. . 2024;11(1):e2043. doi:10.1002/nop2.2043

**Comment 13:** Line 58 typo – “provide” not “provides”

**Response:** Thank you for identifying this error. We have corrected the typo to state “provide”. **Methods:
Comment 14:** Why were private for-profit health facilities excluded? How can you show that the decision to exclude them did not impact the socioeconomic and cultural representativeness of your sample?

**Response:** Thank you for this question. Private for-profit health facilities were excluded from our study due to their differing funding structures and patient demographics, which do not align with the public and not-for-profit facilities more commonly accessed by the broader population. Our focus was on facilities serving diverse socioeconomic groups within the public health system and not-for-profit sector, where discharge practices would likely be most applicable to the majority of patients in the regions studied. To ensure representativeness, we included a range of public and not-for-profit facilities in both urban and rural settings, capturing a diverse cross-section of socioeconomic and cultural contexts.

**Comment 15:** How did you select the health facilities? You said purposively, but using what criteria?

**Response:** Please see our response to Comment 3 where we have addressed this question.

**Comment 17:** How were the 2 districts selected?

**Response:** Thank you for your question. The two districts were purposively selected to implement a district model that integrates referral sites, lower-level health facilities, and community health workers (CHWs) within each district. These districts were chosen based on both their population demographics and healthcare infrastructure. According to the 2014 Uganda National Population and Housing Census, both districts have a high proportion of children under five (16% of the population) and similar access to healthcare, with only 11% of the population living within five kilometers of a health facility. We have updated (bold text) Page 5 to state: “We used both district- and national-level sampling frames to capture a representative sample of the Ugandan health system’s diverse structure and to ensure balanced comparisons across service delivery levels and ownership types. For lower-level health facilities, we selected 3 hospitals and 13 health centres from two districts in Northern and Western Uganda, **chosen for their demographic similarities, including a high proportion of children under five, comparable healthcare access, and varied healthcare infrastructure (Table 1) (Ref). This district-level sampling allowed for a focused examination of local healthcare contexts with shared population characteristics**. **In contrast, to capture the variability of hospitals across Uganda, we used a national-level sampling frame to select 20 hospitals from 20 districts. This broader sampling approach ensured that our hospital sample reflected a wide range of geographical and operational contexts across the country.** Study sites include both public and private not-for-profit (PNFP) ownership types, representing both rural and urban geographical settings. Facilities were eligible for inclusion if they provided pediatric in-patient care and had no prior involvement in the Smart Discharges research program [16]. Private-for-profit health facilities were excluded.”

**Comment 18:** It’s unclear from your methods how you’ve reached the conclusion that your data is nationally representative, given that it excludes representation of lower-level health facilities in large parts of the country.

**Response:** Thank you for your comment. We acknowledge that our sampling of lower-level health facilities from only two districts may limit the full geographic representativeness of these facility types. Our decision to use a district-level sampling frame for health centres focused on two districts with demographic and infrastructural characteristics that align with much of the Ugandan population, including a high proportion of children under five and limited access to healthcare resources, which we anticipated would reflect typical challenges and practices in similar settings nationwide. To complement this, we included a broader national sampling frame for hospitals to ensure that the hospital-level data covered Uganda’s geographical diversity more comprehensively. While our data may not capture all regional variations, we believe this mixed approach provides a balanced overview of key practices and challenges across different facility types. Please see our response to Comment 17 where we provide an update to the methods section regarding sampling frames.

**Comment 19:** You say that caregivers provided written informed consent - did you exclude limited-literacy caregivers, or did you provide them support in order to participate?

**Response:** No, it did not limit enrollment as consent could be provided through either a signature or thumbprint, which is the common practice advised by the local IRB. Consent forms were translated into 11 of the most spoken languages in these regions. We have updated Pages 10-11 to state (new text in bold: “Written informed consent, **obtained through either a signature or thumbprint**, was obtained from individuals participating in the Caregiver and Health Worker Satisfaction Questionnaires, as well as their parent or guardian if the participant was a minor**. Consent forms were available in commonly spoken local languages in each region as well as English**

**Comment 20:** Please further describe any criteria you used to purposively select your health worker participants.

**Response:** Thank you for this comment. With respect to the purposive sampling of health workers, we have described this in detail as being based on “on their level of experience and through consultations with site leads”. Ultimately, we did rely on the relational exposures of the research assistant to both the administrative leadership and the health workers themselves. We believe the somewhat subjective nature of this is captured in our description and if the reviewer or editor wish any additional changes, we would appreciate some specific suggestions which they feel would add the necessary clarity desired.

**Comment 21:** Line 150, 153, 156: closed-ended, not close-ended

**Response:** Thank you. These have all been corrected to “closed-ended”.

**Comment 22:** Not sure that “n” is being used appropriately to refer to the number of items in the score -was each item worth one point? I believe that “n” should be the number of records assessed, and the scoring system should be described separately**.** Is “points” the unit on the median and IQR? There is also variability in how “n” is being used that should be cleared up.

**Response:** We apologize for this discrepancy. We have made changes throughout the manuscript to ensure appropriate use of ‘n’. We have also revised the Statistical analysis section to better describe the scoring system for domain scores.

**Comment 23:** No dash between the number and “items.”

**Response:** Thank you. This is now corrected.

**Comment 24:** Qualitative methods are inadequately described (how were codes determined? How many coders? Who were they? What was the reconciliation process?)

**Response:** Thank you for your feedback. We have removed the qualitative components from this manuscript and aim to report these findings in a separate manuscript, as suggested by several reviewers.

**Comment 25:** The sample size may not be big enough, but it would be helpful to separate out each level of care into private vs. public, to see if different patterns emerge between types of facilities when looking at different levels of the health system.

**Response:** Thank you for your suggestion. The sample size is not sufficient to draw meaningful comparisons between private and public facilities at each level of care without introducing potential biases or limiting the generalizability of our findings.
 **Results:
Comment 26:** I generally found the results difficult to follow due to unclear phrasing, excessive passive voice, and difficulty differentiating among the different tools used and what their various purposes were. There is also widely variable and inappropriate use of “n” to describe various numbers. Generally this section needs to be edited to the most important findings; the authors might consider including others in a second manuscript.

**Response:** Thank you for your feedback. We have revised the results section to reduce passive voice, clarify the use and purpose of the different tools, and ensure consistent and appropriate use of "n." Additionally, we have streamlined the section to focus on the most important findings; however, we have opted to provide comprehensive results in this manuscript as we justified above.

**Comment 27:** What does “routinely discharged” mean? (line 196).

**Response:** A routine discharge refers to the standard process of releasing a patient from a healthcare facility when they are deemed medically stable and no longer require inpatient care. The remaining 2% of patients were either discharged against medical advice (1.4%) or referred to a higher level of care (0.7%), as shown in Figure 1.

**Comment 28:** Why is the only demographic information reported for the caregivers the distance they travelled to the hospital/health centre? Were other characteristics collected? If so, these would help us understand the socioeconomic and other demographics of the population studies, which is likely to be important, especially when determining whether this is truly a nationally representative sample.

**Response:** Thank you for your comment. We specifically selected health facilities that are reflective of the Ugandan population, particularly those that serve individuals who access public or private-not-for-profit (PNFP) facilities, which tend to cater to lower-income communities. While we did not collect additional demographic or sociodemographic data as it was not part of the purpose of the facility readiness scan, we recognize the value of such information. We can provide this feedback to the Sepsis CoLab for consideration in revising the tools for future studies.

**Comment 29:** Pretty impressive that 80% of facilities provided meds for free. Are meds supposed to be free by law or policy in the public system in Uganda?

**Response:** Yes, public facilities in Uganda are supposed to provide medications for free. However, due to a lack of resources, many do not have medications available so patients are forced to purchase out-of-pocket, as seen in our results – 96% of public facilities experience stock outs. We updated the methods section (Line 86) to state: “In Uganda, public facilities offer health services and medications at no cost, while fees vary across private facilities.”

**Comment 30:** Line 217: I can’t follow what this line means: “Facilities having referral clinics for 218 post-discharge follow-up was more commonly reported among hospitals (56.5%, n=13) than 219 health centres (7.7%, n=1) (p=0.011) and PNFP facilities (50%, n=4) compared to public 220 (35.7%, n=10) (p=0.683).”

**Response:** We apologize for the lack of clarity. This sentence has been updated to provide more clarity stating: “Hospitals reported having referral clinics for post-discharge follow-up more often (56.5%, n=13) than health centres (7.7%, n=1) (p=0.011).” and removed the ownership outcome as it is not significant.

**Comment 31:** Results should be formatted: (median = 3; IQR = 2 – 4) or simply (m=3, IQR=2-4)

**Response:** Thank you. This has been corrected throughout the results section.

**Comment 32:** Line 34: typo “owrnership”

**Response:** Apologies for this typo. The spelling error is now corrected.

**Comment 33:** Line 241: “Although socio-economic history taking was not commonly performed and documented among all cases, domain scores (n=7 items) were significantly higher (p<0.001) among health centres (median 4: IQR: 2 – 4) compared to hospitals (median 1; IQR: 0 – 2) (S2 Fig, S3 Table).,” can be simplified to something like: “Socio-economic history was infrequently documented overall. However, in health centres the median socio-economic history-taking score was 4 (IQR=2-4), higher than the median score at hospitals (median=1, IQR=0-2) (S2 Fig, S3 Table).”
Related note: how do you know that indicators were infrequently performed? Does the score reflect both whether the health care worker took the history and also whether it was documented? What’s the significance of including both of those measures?

**Response:** Thank you for this suggestion. Lines 248-251 have been updated to clarify the point that “Although health workers in general did not commonly perform and document Socio-economic History Taking, health centres had significantly higher scores in this domain (7 items) (m=4; IQR=2-4) than hospitals (m=1; IQR=0-2) (*p*<0.001) (S2 Fig, S3 Table).” When we say "infrequently performed," we mean that we did not observe health workers performing and documenting these variables consistently across all cases. Our data collection focused on whether health workers both “performed AND documented” these variables, rather than just performing them. We reviewed the manuscript, tables, and figures to ensure that these variables clearly state “performed and documented” We did not assess instances where the assessment was performed but not documented, as we considered that failing to document the assessment in the patient record would undermine its potential to influence care, particularly regarding continuity of care between health workers.

**Comment 34:** Line 332: “insignificant” should be “not significant.” Also, if it is not statistically significant, it is inaccurate to say that it was higher. You could say that it “trended toward” being higher, though that’s also usually not advised, because the statistical analysis does not support it.

**Response:** Thank you for your feedback. We have removed this statement from Line 332. We have reviewed the results section and updated the language used to say either trended toward or removed the statements of higher or lower when not significant.

**Comment 35:** It would be helpful to describe qualitative themes more systematically. You may also need a frequency table for codes or themes, or a weighting schema in your analysis, since you report that several things are “commonly reported” but don’t explain exactly what you mean by that, or which were the most prominent themes in your analysis.

**Response:** Thank you for your feedback. We have removed the qualitative components from this manuscript and aim to report these findings in a separate manuscript, as suggested by several reviewers.

**Comment 36:** Line 340: “Health workers were asked to rate staff availability for several discharge scenarios at their facility.” This belongs in the methods, along with richer description of what the various scales are assessing.

**Response:** Thank you. We have removed this line form the results and revised the methods section to state that the satisfaction questionnaires included closed-ended questions and Likert scales (Line 166). **Discussion:
Comment 37:** Line 352: Incomplete sentence: “This is not surprising and in view of the high mortality rates post-discharge.”

**Response:** Our apologies. The statement has been updated as follows: “Our study found that discharge related resources and care practices were suboptimal across the facilities studied, which is not surprising given the high post-discharge mortality rate among children under 5 years in Uganda.”

**Comment 38:** Lines 384-385: It’s unclear to me how health workers doing something that isn’t part of the formal discharge process supports the importance of job aids and education in discharge planning.

**Response:** Thank you for your comment. The observation that health workers at health centres sometimes took the initiative to schedule follow-up referrals, even when it wasn’t part of the formal discharge process, highlights the value of close community relationships. However, this also underscores a gap in the formal discharge procedures and the potential for inconsistency in discharge practices. Providing job aids and comprehensive training could help standardize and support discharge planning across facilities, ensuring that all health workers have the necessary guidance and tools to adhere to best practices consistently, regardless of informal community connections.

We have updated this paragraph to better describe this connection: Resources aside, observing discharge practices provides valuable insight into facility performance. We observed discrepancies between observed and reported discharge practices across all settings. Both hospital and health centre workers did not consistently adhere to their respective facility's reported discharge procedures, such as providing discharge counselling, take-home materials, and scheduling follow-up referrals. Interestingly, some health workers took the initiative to scheduled follow-up referrals even though it was not officially reported as a key discharge practice at their facility. This behaviour may reflect the strong community relationships often seen at health centres [25]. These findings underscore the need for standardized discharge practices and highlight the importance of job aids and discharge training to ensure all health workers can consistently adhere to evidence-based discharge protocols, regardless of informal community ties.

**Comment 39:** Line 393: What “cultural differences?”

**Response:** Thank you for your comment. The term "cultural differences" in this context was meant to refer to the variations in care delivery practices, patient-provider interactions, and community expectations that can exist between different types of health facilities (e.g., health centres vs. PNFP facilities) and the communities they serve. These differences can impact caregiver perceptions of care and satisfaction. We acknowledge that this could benefit from further clarification and will adjust the language to provide more specific examples or context, if necessary. We updated this statement (new text in bold) as follows:

“Several factors such as staffing inadequacy as well and cultural differences, **such as variations in care delivery practices, patient-provider interactions, and community expectations**, between service delivery levels may account for these findings and warrants further exploration.”

**Comment 40:** Separate out limitations in a new sub-section.

**Response:** Thank you. We have separated out limitations into a sub-section.

**Comment 41:** Add “strengths” sub-section.

**Response:** This is an excellent suggestion. A Strengths section has been added to Page 24.

“This study has several key strengths. It utilized a nationally representative sample of health facilities, providing a comprehensive overview of discharge care across diverse settings in Uganda. Notably, the study incorporated a thorough assessment of resource availability, procedures, and care practices specifically related to hospital discharge, an area that is often overlooked in existing facility assessments. Additionally, the inclusion of both health worker and caregiver satisfaction provides valuable insights into the human factors influencing care, which are frequently neglected in facility assessments. These strengths contribute to a more holistic understanding of discharge processes and their impact on care quality.”

**Comment 42:** This is a big assumption: “However, it is unlikely that differences between districts would vastly exceed differences within districts, and thus, we do not believe that our sampling significantly biased our results as they pertain to country-level inferences.” Is there any data from other studies to back this up and support the generalizability of your findings?

**Response:** Thank you for your thoughtful comment. We have revised the manuscript to clarify this point. In response to your concern, we now state (Pg 27, line 532): " However, it is possible that differences between districts exceed our observations of the differences within districts, though the wide variation within districts suggests that for this to be true, the differences would need to be quite significant. Given this, we do not believe that our sampling significantly biased our results as they pertain to country-level inferences."

**Comment 43:** Discussion of the limitations of the observation component is difficult to contextualize, as the observations aren’t really described, so it’s unclear how big an impact this limitation might have had.

**Response:** We may have not sufficiently described this limitation. Essentially we are describing the hawthrone bias, whereby an observer, merely by being present, changes the behavior of the one being observed. We have adjusted this sentence on page 27, line 538

**Comment 44:** What is a “think-aloud approach?”

**Response:** The think-aloud approach is a method in which “cognitive processes and decision-making strategies by having people voice their thoughts while performing a task or solving a problem.” We have now provided a reference for this definition n the manuscript. Ericsson KA, Simon HA. 1980. Verbal reports as data. Psychol Rev. 87(3):215–251. doi:10.1037/0033-295X.87.3.215

**Comment 45:** If possible, it would help to better characterize COVID-related resource and staffing shortages in these clinics, to help the reader understand the context and limitations of the study better.

**Response:** Though we would desire to specify the staffing issues caused by COVID, we do not have these data. We merely have the reliable, yet subjective knowledge that COVID did cause these kinds of disruptions. We currently state that “this study was conducted during the COVID-19 pandemic which may have impacted resource availability due to supply chain issues as well as satisfaction with discharge care due to staffing shortages, potentially introducing bias into our results.”

**REVIEWER #3:**

**Comment:** Overall, this is a thorough look at discharge practices in selected health centres and hospitals in Uganda. The authors clearly worked hard to gather the data presented here. However, there is a lot presented. This manuscript could benefit from some editing to make everything more concise, or by removing some of the findings to write them up separately. Specific comments by section below:

**Response:** Thank you for your thoughtful feedback and recognition of the effort we put into gathering and presenting the data. We have made edits to enhance the conciseness of the manuscript, focusing on streamlining the presentation of findings. Additionally, we have considered your suggestion to remove some findings for separate analysis and have adjusted the manuscript accordingly and have indicated where we made these changes in our responses below. Specifically, the qualitative components from this manuscript have been removed as we aim to report these findings in a separate manuscript, as suggested by several reviewers. We appreciate your guidance in this process and believe these changes strengthen the overall clarity and impact of our work.
 **Abstract
Comment 1:** Results: can you give more info on “infrastructure domain scores” or use the same language as in the methods?

**Response:** Thank you for this comment. We have updated that line in the abstract to state “Infrastructure availability was significantly higher among hospitals compared to health centres (p<0.001) with no difference between public and private-not-for-profit facilities.” **Intro
Comment 2:** In line 52, I think it should be “sub-Saharan Africa” given the abbreviation that follows.

**Response:** Thank you for identifying this type. Line 52 has been updated accordingly.

**Comment 3:** The WHO info (lines 67-70) is good information but maybe not placed well; consider moving it to the 2nd paragraph, perhaps around line 61.

**Response:** Thank you for this suggestion. We updated the paragraph to connect the WHO guidelines to our study aim in response to another reviewer’s feedback. We hope that you feel the placement seems more relevant with these revisions.

**Methods
Comment 4:** You give a lot of background in the types of health centres/hospitals in Uganda, then simply combine them into health centres and hospitals. You could simplify this paragraph somewhat since the division of type of health centre or hospital isn’t used in analysis.

**Response:** This is a great suggestion, thank you. We’ve revised this paragraph to be more concise (Page 5, lines 82-85).

**Comment 5:** I’m not sure I understand the district vs national sampling used. Are there more lower level health centres in those 2 districts and that is why more were chosen from 2 districts? Is the population different in those 2 districts vs the other 20? A bit more explanation would be helpful.

**Response:** We apologize for the lack of clarity. We have updated page 5 to provide more explanation, stating “We used both district- and national-level sampling frames to capture a representative sample of the Ugandan health system’s diverse structure and to ensure balanced comparisons across service delivery levels and ownership types. For lower-level health facilities, we selected 3 hospitals and 13 health centres from two districts in Northern and Western Uganda, chosen for their demographic similarities, including a high proportion of children under five, comparable healthcare access, and varied healthcare infrastructure (Table 1). This district-level sampling allowed for a focused examination of local healthcare contexts with shared population characteristics. In contrast, to capture the variability of hospitals across Uganda, we used a national-level sampling frame to select 20 hospitals from 20 districts. This broader sampling approach ensured that our hospital sample reflected a wide range of geographical and operational contexts across the country. Study sites include both public and private not-for-profit (PNFP) ownership types, representing both rural and urban geographical settings. Facilities were eligible for inclusion if they provided pediatric in-patient care and had no prior involvement in the Smart Discharges research program [16]. Private-for-profit health facilities were excluded.”

**Comment 6:** Under participant sampling and recruitment: is there a reason enrollment was limited to those admitted for suspected sepsis?

**Response:** Dr Tedros Adhanom Ghebreyesus, the WHO Director-General, has stated that sepsis care is the bellwether of the quality of care in health systems. Therefore, the Sepsis CoLab designed the Environmental Scan tools utilized in this study as a tool to determine readiness for treating sepsis knowing that overall care of critically ill children in the facility will be improved by quality improvement initiatives to improve pediatric sepsis care. We have made the following revisions to make this more clear:

Introduction (new text in bold):

“In 2021, over five million children under the age of five died globally, with sub-Saharan Africa (SSA) accounting for 53% of these deaths. **Most of these deaths are due to infectious diseases, with sepsis being the leading immediate cause of death in sub-Saharan Africa and South Asia**. Uganda has one of the highest under-5 mortality rates in SSA at 58.4 deaths per 1,000 children. An important, but neglected, aspect of child mortality are deaths occurring during the post-discharge period which often exceed the burden of in-hospital deaths.

We have also provided additional references to support our point that sepsis, and infectious diseases more generally, are a leading cause of preventable deaths among under-five children in developing countries; in particular, Rudd et al. 2020 report that sepsis is implicated 1 in 5 deaths globally and children under 5 account for 40% of cases and 2.9 million deaths.

*Bassat Q, Blau DM, Ogbuanu IU, et al.: Causes of Death Among Infants and Children in the Child Health and Mortality Prevention Surveillance (CHAMPS) Network. JAMA Netw Open 2023; 6:e2322494*

*United Nations Inter-Agency Group for Child Mortality Estimation (UN IGME): Levels and trends in child mortality [Internet]. 2023; [cited 2024 Mar 14] Available from:* [*https://data.unicef.org/resources/levels-and-trends-in-child-mortality-2024/*](https://data.unicef.org/resources/levels-and-trends-in-child-mortality-2024/)

*Rudd KE, Johnson SC, Agesa KM, et al.: Global, regional, and national sepsis incidence and mortality, 1990-2017: analysis for the Global Burden of Disease Study. Lancet (London, England) 2020; 395:200–211*

Methods (new text in bold):

All study sites underwent a 5-survey Facility Scan informed by the Pediatric Sepsis Data CoLaboratory’s (Sepsis CoLab) Environmental Scan [17] which is used to support health facilities in identifying quality improvement priorities in **treating children with sepsis.**

**Comment 7:** Did the ability to provide written informed consent limit enrollement? What language was the informed consent document written in?

**Response:** No, it did not limit enrollment as consent could be provided through either a signature or thumbprint, which is the common practice advised by the local IRB. Consent forms were translated into 11 of the most spoken languages in these regions. We have updated Pages 10-11 to state (new text in bold: “Written informed consent, **obtained through either a signature or thumbprint**, was obtained from individuals participating in the Caregiver and Health Worker Satisfaction Questionnaires, as well as their parent or guardian if the participant was a minor**. Consent forms were available in commonly spoken local languages in each region as well as English.**

**Comment 8:** The number of different scores used (lines 161 onward) gets somewhat confusing, if there is any way to simplify this**.**

**Response:** Thank you for this comment. We have attempted to simplify the domains score grouping in methods section (Pages 9 & 10).

**Comment 9:** Were the observational surveys done on the same children that caregivers completed surveys about?

**Response:** Observational surveys and caregiver surveys were often conducted concurrently on different children to manage data collection time efficiently; responses could not be linked across surveys as patient identifiers were not collected. We added the following statement to the Study Procedures to clarify this (Line 116): “Caregiver Satisfaction Survey participants were not necessarily the same as those included in the Observational Scan.”

**Results
Comment 10:**  Line 196, What do you mean by “routinely discharged”? What happened to the other 2%?

**Response:** A routine discharge refers to the standard process of releasing a patient from a healthcare facility when they are deemed medically stable and no longer require inpatient care. The remaining 2% of patients were either discharged against medical advice (1.4%) or referred to a higher level of care (0.7%), as shown in Figure 1. To keep the manuscript concise, we chose not to include these specific details within the main text.

**Comment 11:** Line 213-214, you mention the 29 provided for free and 8 provided meds at cost; that doesn’t add up to 36. Was there overlap where some meds were free and others at cost?

**Response:** Apologies for that typo. We have corrected this to 7 provided meds at cost (Line 233).

**Comment 12:** Line 217, the % and N for the public facilities doesn’t make sense; if it’s 96% of them with stock outs then the n should be higher than 1.

**Response:** Thank you for identifying that typo. The n has been corrected to n=27 (96.4%).

**Comment 13:** For the admission section, if there are 180 observations, is it 180 unique patients or unique observations? Was each patient observed at admission and discharge to comprise 1 observation?

**Response:**  Yes, each patient observed at admission and discharge to comprise 1 observation. See Lines 160-162 which have been updated to state “The Observational Scan (Survey 3) involved a research assistant observing health workers throughout the admission and discharge process for patients who received in-hospital treatment for severe infection, comprising a single observation per patient.”

**Comment 14:** Line 299, you report that 19.9% of health centre cases were not provided with take-home materials, but in table 2 you report that only 4% received take home materials, so this doesn’t add up; the numbers for hospitals also don’t add up.

**Response:** Thank you for identifying this error. We have corrected the statistics as follows “caregivers were not provided with take-home materials in 64.3% of hospital cases and 93.8% of health centre cases,”

**Comment 15:** Overall results section is very lengthy and could benefit from trimming down some of this information or perhaps even dividing it into 2 manuscripts (the survey and observation I one, the caregiver/health care worker satisfaction in another). The high number of tables and figures make it difficult to follow the text sometimes.

**Response:** Thank you for your feedback. We have carefully reviewed and edited the results section to reduce its length. We have also removed the qualitative components from this manuscript and aim to report these findings in a separate manuscript, as suggested by several reviewers.

**Discussion
Comment 16:** Again, all the different names for the different surveys/scans gets confusing (line 353).

**Response:** This has been revised to state “Assessments of resources and infrastructure revealed…” rather than “the Environmental and Technology Scans revealed…” for clarity.

**Comment 17:** The results section spends a lot more time looking at the care practices/satisfaction than the resource availability, but the discussion spends more time looking at resources and ways to scale up resources (makes sense that improving resources could improve the rest of it, but the comment in line 375 seems to indicate that resources are less important).

**Response:** Thank you for this observation. We have made changes throughout the discussion and results sections to more clearly and succinctly align the connections between care practices, satisfaction, and resource availability. Specifically, we have emphasized how both resource availability and care practices contribute to the overall quality of discharge care.

**Comment 18:** Can you comment on the disconnect between reasonably high satisfaction levels but low overall discharge preparation/guidance?
- Appropriate discussion of limitations

Thank you for this comment. We agree that this was an interesting observation that nevertheless requires further investigation. We have attempted to bring this issue into the discussion in several ways, but feel the manuscript is already quite lengthy and are thus reluctant to further discuss this issue. We are continuing to do this kind of work within the East African context and will undoubtably investigate this issue in more depth in the coming years.

On this issue we currently state the following:

Page 20: “The job aids may contribute to the higher health worker satisfaction with the discharge process at health centres when compared to hospitals of all types. However, other factors such as a closer interpersonal relationship between healthcare members and family members and others within the community may also account for greater satisfaction by fostering a collaborative work environment and a sense of trust [24,25].”

Page 21: “An important component of care delivery in any setting is the satisfaction of both the givers and recipients of care [28]; however, health worker and caregiver perspectives are often overlooked in facility assessments [29,30]. Our findings revealed higher satisfaction at health centres and PNFP facilities as well as high levels of satisfaction with counselling content when discharge education was received, suggesting that discharge counselling may improve overall caregiver satisfaction. Health workers also recognized the need to improve counselling through various means including providing caregivers with take-home materials and establishing counselling guidelines. Several factors such as staffing inadequacy as well and cultural differences between service delivery levels may account for these findings and warrants further exploration. Caregiver opinions matter in that they are more likely to adhere to follow-up if they perceive that they are being treated with dignity and respect [28]. Moreover, caregiver satisfaction is associated with care coordination and family engagement interventions, such as scheduling follow-up care, written care plans, and parental education [31]. “

**Response:

Conclusion
Comment 19:** Your results seem to argue more that health centres provide better counselling while hospitals do better with materials, risk assessment, and follow up referrals which doesn’t necessarily mean that health centres do better with discharge care overall (although satisfaction levels imply they may)

**Response:** This is an appropriate observation and one we have attempted to highlight. We feel that this information will be valuable in efforts to develop methodologies in improving discharge care within a Ugandan (perhaps more widely) context. **Tables
Comment 20:** Overall, there are way too many tables and figures in this manuscript. Is there any way to combine or remove some of them? The supplemental figures are referenced pretty frequently without a lot of description, and it can sometimes be difficult to find supplemental figures after publication
- You could combine S2, S3, S4, S5, S6 much like S1 or remove some of those and combine the rest
- S3 table, S4 table also appear to be in another form elsewhere and therefore repetitive
- S7, S8 are included in Table 2 and not necessary
- S1 Table is less informative than S2 Table
- Table 1; if you simplify some the description this could be a supplemental table if it’s used at all, freeing up a space for another results table
- Table 2, under discharge documents provided, n for discharge form for hospitals is 41, but only 40 in the next section of the table

**Response:** Thank you very much for these suggestions. We have reduced the number of tables and figures accordingly to ensure clarity and ease of navigating the manuscript. Please see the supporting information (Pg 29) for a list of revised supplementary materials.
