# Supplementary material for: Discharge policies and care practices for children with suspected sepsis: A health facility scan at a nationally representative sample of hospitals and health centres in Uganda
Source: PLOS Glob Public Health. 2025 Aug 22;5(8):e0003559. doi: 10.1371/journal.pgph.0003559 (PMC12373204; doi:10.1371/journal.pgph.0003559)
Supplement: Attachment — Submitted filename: FScan_Response_to_reviewers_2_FINAL.docx [file pgph.0003559.s004.docx]

**RESPONSE TO REVIEWERS**

**Manuscript reference number:** PLOS GLOBAL PUBLIC HEALTH PGPH-D-24-01608

**Title:** Discharge policies and care practices for children with suspected sepsis: A health facility scan at a nationally representative sample of hospitals and health centres in Uganda

June 13, 2025

To the Editor,

Thank you for allowing us to continue to improve this paper. We have taken these reviews very seriously and have made substantial changes to further improve this manuscript. Specifically, we have re-organized our methods and results sections substantially to address the issues of clarity that have persisted during this revision process, and we hope that further clarity has indeed been added. Given the substantial changes to these 2 sections, we have not fully captured all changes in the specific responses as we usually do in responding to reviewer comments (i.e. quoting the new text addition and removal), as this may add further confusion in reviewing these changes. Rather, we have, to the best of our ability, responded to each comment to clarity and have pointed out where in the manuscript this issue has been addressed (line numbers in the clean version, which will be simpler to review).

We hope that these changes now make our paper suitable for publication in PLOS Global Public Health.

Best wishes,

Matthew O. Wiens, on behalf of all co-authors

Reviewer's Responses to Questions

**Comments to the Author**

 1. If the authors have adequately addressed your comments raised in a previous round of review and you feel that this manuscript is now acceptable for publication, you may indicate that here to bypass the “Comments to the Author” section, enter your conflict of interest statement in the “Confidential to Editor” section, and submit your "Accept" recommendation.

Reviewer #1: (No Response)

Reviewer #2: (No Response)

2. Does this manuscript meet PLOS Global Public Health’s [publication criteria](https://track.editorialmanager.com/CL0/https:%2F%2Fjournals.plos.org%2Fglobalpublichealth%2Fs%2Fcriteria-for-publication/1/010f01961011fb91-209bac16-c7b3-40c6-8daf-1b53d06d137d-000000/FwGbh-GZBoKsGrwsOg5zvjbIGhhR5tdcWezNK5my7No=205)? Is the manuscript technically sound, and do the data support the conclusions? The manuscript must describe methodologically and ethically rigorous research with conclusions that are appropriately drawn based on the data presented.

Reviewer #1: Partly

Reviewer #2: Yes

3. Has the statistical analysis been performed appropriately and rigorously?

Reviewer #1: Yes

Reviewer #2: Yes

4. Have the authors made all data underlying the findings in their manuscript fully available (please refer to the Data Availability Statement at the start of the manuscript PDF file)?

 The [PLOS Data policy](https://track.editorialmanager.com/CL0/https:%2F%2Fjournals.plos.org%2Fglobalpublichealth%2Fs%2Fdata-availability/1/010f01961011fb91-209bac16-c7b3-40c6-8daf-1b53d06d137d-000000/-KW9D3_01l93zmKGTYEtFNm3rwSU-jyIl-kIqReHsyY=205) requires authors to make all data underlying the findings described in their manuscript fully available without restriction, with rare exception. The data should be provided as part of the manuscript or its supporting information, or deposited to a public repository. For example, in addition to summary statistics, the data points behind means, medians and variance measures should be available. If there are restrictions on publicly sharing data—e.g. participant privacy or use of data from a third party—those must be specified.

Reviewer #1: Yes

Reviewer #2: Yes

5. Is the manuscript presented in an intelligible fashion and written in standard English?

 PLOS Global Public Health does not copyedit accepted manuscripts, so the language in submitted articles must be clear, correct, and unambiguous. Any typographical or grammatical errors should be corrected at revision, so please note any specific errors here.

Reviewer #1: Yes

Reviewer #2: Yes

6. Review Comments to the Author
Please use the space provided to explain your answers to the questions above. You may also include additional comments for the author, including concerns about dual publication, research ethics, or publication ethics. (Please upload your review as an attachment if it exceeds 20,000 characters)

**REVIEWER #1:**

**Comment:** This manuscript assesses the readiness of health facilities in Uganda to manage pediatric discharge care for children under 5 years or age, focusing on 36 representative health facilities. The authors have made significant revisions in response to reviewer comments, which have strengthened the paper.
Due to its scope and design, as well as its potential to impact practices in LMICs, it continues to be a strong candidate for publication.
 The manuscript would benefit from improved organization and consistency in reporting the outcomes, which would make it easier for readers to follow significant findings.

**Response:** Thank you for recognizing the potential impact of our study and for noting the significant revisions made in the previous round. In response to your feedback, we have further reviewed and improved the organization and consistency in reporting the outcomes to enhance clarity and readability.

**Recommendations:**
**Comment:** Abstract: Ensure consistency between the abstract methods, results, and conclusion and the manuscript in terms of primary and secondary outcomes, and findings based on those outcomes.

**Response:** Thank you for highlighting the importance of consistency between the abstract and the main text. We reviewed the abstract and updated it where necessary to ensure alignment in the reporting of primary and secondary outcomes, as well as the key findings.

***Methods***:

**Comment:** Line 84: Consider including total N for hospitals in Uganda, as the total number of admitting facilities is provided.
**Response:** We have now revised the text to include the total N for hospitals. **Lines 86 and 87**

**Comment:** Consider clarifying the difference in services between health centres and hospitals, as both types of facilities admit patients (unlike in the United States, where typically only hospitals admit). This distinction could be in the methods or introduction.
**Response:** Thank you for this helpful suggestion. We have clarified this on **Lines 85 and 86**.

**Comment:** Was immunization and record keeping considered, as it is noted as part of the four key WHO guidelines for effective hospital discharge? If not, this could be addressed in the discussion or limitations section.
**Response:** Thank you for this comment. Yes, immunization status and record keeping were assessed as part of the observed health worker care practices at both admission and discharge. Please see S2 Table. *Observed health worker care practices at admission* and Table 2, *Comparison of observed discharge practices between hospitals and health centres,* as both include details on whether immunization status, among others, was documented.

**Comment:** Please provide further clarification on how the primary outcomes were determined. Specifically, how was the resource availability score calculated? How was observation of the four key discharge care practices quantified? How are resource availability and observation scores combined to determine overall facility readiness for discharge? It would also be helpful to explain the rationale for why these indicators were chosen to represent the facility readiness for discharge score. It would also be helpful to clarify what defines a good outcome for facility readiness for discharge.
**Response:** Thank you for this thoughtful comment. We have substantially revised the way we explain the outcome. Please review the updated methods section (**Lines 140-152**). I hope further clarity has been added to avoid confusion.

One minor change (beyond adding clarity) to how we reported our primary outcome, is that we have now included the key practice of “clinical assessment on the day of discharge” to the primary outcome, alongside the other for items. This item was already listed as a key discharge practice, but since the facility survey did not include this (it was only included in the observational scan of patients being discharged), the comparison between observed (at the individual level) and reported (at the facility level) could not be made. See **lines 147-150**, where we state: *“The practice of conducting a clinical assessment on the day of discharge was not compared in this manner as it was assumed all facilities would report this as being necessary”*. We believe how we have clarified our outcomes will now make more sense to readers and reviewers.

**Comment:** There is some inconsistency in the description of the primary outcome. In the study outcomes section (Lines 146-147), facility readiness for discharge is defined as both resource availability and observation of discharge practices. However, in the statistical analysis section (Lines 170-175), it states that facility readiness scores are calculated based solely on resource availability. Please clarify which of these is accurate
**Response:** Our revised methods section will hopefully have provided more clarity on this issue.

**Comment:** The methodology for calculating care practice domain scores from the seven domains needs further clarification, as well as how these relate to the four key discharge care practices listed in the primary outcomes. It would be helpful to explicitly outline this for the reader.
**Response:**  Thank you for this comment. We hope we have now added sufficient clarity. See **lines 154-171**

**Comment:** Clarify how the secondary outcome of observed versus reported discharge care practices is calculated and how it differs from the primary outcome of “observation of four key discharge care practices.”
**Response:** Thank you for this comment. We hope our revised methods has added the necessary clarity.

***Results:***

**Comment:** Consider presenting results of the primary outcome, facility readiness for discharge, before breaking it down into its components.

**Response:** We apologize for this confusion. we have revised how we report the primary and secondary outcomes. Please review the full results section, as several sections have been adjusted to add clarity.

**Comment:** Consider presenting the results of overall resource availability scores before breaking them down into individual domains (e.g., infrastructure, technology, forms & job aids, equipment). This would help with the interpretability of the results.
**Response:** See the above response.

**Comment:** Similarly, present the overall care practice scores before detailing the breakdown of specific practices.
**Response:** Similar to the Facility Readiness scores, we did not calculate an overall care practice score.

**Comment:** Consider restructuring Table 2 to align more clearly with the primary outcomes. Currently, it takes time for the reader to align the subheadings in the table with either the four key discharge care practices or the seven domains presented in the statistical analysis.
**Response:** We hope that our improved results sections clarifies how the tables can be integrated while reading the main text.

***Discussion:***

**Comment:** Consider summarizing all pertinent findings regarding the primary outcomes in the first paragraph before discussing the individual components. This would provide clarity for the reader and highlight key findings early in the discussion.
**Response:** Thank you for this suggestion. We have added the necessary clarity on this.

**Other comments:**
**Comment:** In Table 2, consider highlighting significant p-values (for example, by putting an asterisk or bolding them)
**Response:** Thank you. An asterisk has been added to signify statistical significant p-values.

**Comment:** Consider copy-editing for minor spelling errors. For example, Line 279 “In contract”, should be “In contrast.”
**Response:** Thank you for identifying this spelling error. We have done a full copy edit and corrected any remaining errors.

**Comment:** Overall, this manuscript provides valuable insights into pediatric discharge readiness in low and middle income countries and has the potential to make a substantial contribution to the literature.
**Response:**Thank you for this encouraging feedback. We appreciate your recognition of the manuscript’s potential contribution to the literature on pediatric discharge readiness in low- and middle-income countries.

**REVIEWER #2:**

**Comment:** I appreciate that the authors have spent significant time revising the manuscript, and the overall study is extremely worthy and very interesting, shedding light on an important and oft-overlooked element of care. They have addressed many of my previous comments and overall the paper is much improved and easier to read. In my opinion, though, it is still not quite ready for publication, primarily because the many scores, percentages and referenced tools still make it difficult to follow, and the references to supplementary tables are still too frequent. I needed to have two separate pdfs of the manuscript open in order to read it, because following all the details required so much scrolling and re-reading, especially when reviewing the results section.

I would suggest that the writing team use their primary tables and figures (which should present the most important findings) to guide the writing, and either provide all other data in a set of comprehensive supplementary tables that are NOT necessary to reference to understand the main results, or separate into several manuscripts. The presented findings should also correlate to the specific study objectives, with very sparing presentation of interesting findings from individual questionnaires or response fields.
**Response:** Thank you for your kind feedback and for recognizing the improvements made. We truly appreciate your thoughtful suggestions, which have strengthened the clarity of the manuscript. In response to your comment, we have moved Table S1 into the main manuscript (now Table 2) to improve flow and reduce reliance on supplementary materials. We also reviewed the writing to better align the results with our primary tables and study objectives.

**Further comments by section:

ABSTRACT:**
**Comment:** Study objectives are not presented as presented in the writeup; please include these, rather than describing the facility scans, as the facility scans have been broken down (as I understand it) in a different way to answer the study questions.
**Response:** The study objectives are nearly verbatim in the abstract (Lines 25-28) as mentioned in the body of the manuscript (Lines 75-77). However, we did include the primary outcomes and revised the results section of the abstract to align more closely with what is reported in the main manuscript. We hope this addresses your valuable feedback.

**Comment:** Why is the p value for post discharge counselling presented as <0.021? Is this an error?
**Response:** Our apologies for that error. It has been corrected to p=0.021.

**INTRODUCTION:
Comment:** Consider introducing the Environmental Scan and its previous uses in the introduction, rather than discussing the WHO guidelines, which are never mentioned again, and it's unclear how exactly they are related to the scan used in the methods.
**Response:** We appreciate your suggestion. However, we chose to include the WHO Guidelines for the Management of Common Childhood Illnesses in the Introduction to provide a globally recognized benchmark for pediatric discharge care. These guidelines highlight core discharge components that align with our study’s focus and were used to conceptually frame the relevance and urgency of evaluating discharge practices in Uganda. While the Environmental Scan was introduced later as the specific methodological tool, its content was adapted to assess the discharge components emphasized by WHO. Therefore, we believe the current structure provides a logical and globally grounded rationale for the study’s focus and helps readers understand the importance of these elements in the broader child survival context. We respectfully maintain the current structure of the Introduction.

**METHODS:
Comment:**  Sampling description is much improved! Thank you.
**Response:** Thank you for your positive feedback. Some of the sections on sampling and the facility scan have been shorted with futher details added to the supplement for those interested in this. We hope this has added clarity for reviewers and readers alike.

**Comment:** The authors responded in the comments to reviewers as to why for-profit hospitals were excluded but still do not detail the reason for this exclusion in your methods, which I would argue does need to be addressed, just with a one-sentence explanation.
**Response:** We apologize for not addressing this in the last round of edits. We’ve updated the text (bold) to state “ Private-for-profit health facilities were excluded “*due to differing funding models and patient populations, which are less representative of the broader public and not-for-profit sectors serving most Ugandan children***.**” (**Lines 91-93**).

**Comment:** Would move description of your data collection tools above your participant recruitment, because you currently reference the questionnaires before you tell us what questionnaires you use, and it reads as out-of-order.
**Response:** Thank you for this thoughtful suggestion. we have adjusted our methods significantly and we hope clarity has been added.

**Comment:** Thank you for more fully describing the Sepsis CoLab environmental scan. This reads as though informal consultation was the only method used to write discharge modules. Understanding that this may be the case and may be related to resource or time constraints, if there were more formal methods (focus groups, delphi method, etc) used to write these modules, they should be included here (lines 136-143) or referenced if published elsewhere.
**Response:** Thank you for this question. No formal methods beyond what is described were used for developing the discharge modules. However, the full methodology for the development and validation of the Environmental Scan tools is detailed in the cited references.

**Comment:** The elements of the surveys that are used to generate each outcome are not a statistical method, and so in my opinion should be described in your Study Outcomes section, not your statistical analysis section. This would also help clarify the relationship between your outcome measures and your study instrument. This clarification might also be achieved by just breaking up your paragraphs more, and starting each with a topic sentence that reads "xxx outcome measure was calculated using xxx scans." But also, methodologically is it actually important which scan each of the measures comes from? Would it be possible to just say "We compiled scores using items from the 5-survey Facility Scan..." without going into so much detail about the scans themselves, especially in the statistical methods? Does it matter which survey the measure comes from, as--as I understand it--you recombined them all anyway? Think about it as treating the Environmental Scan as one tool rather than a set of 5 tools. You can include the details in a supplement because they are not essential for understanding your outcomes, and so if the reader can't or doesn't want to access them, it's ok.
**Response:** We have adjusted our analysis section to better fit the required content. Since the survey is site specific, we were required to consider both the analysis of the survey (compiling survey results) as well as analysis of the outcomes. We have made adjustments to this section, including renaming this sub heading as “*Survey and statistical analysis*” We hope clarity has been added (**Lines 154-184**).

**Comment:**  I do not understand this line: Missing data were removed from the analysis and reported throughout the manuscript as numerators over denominators to clarify the variables affected by missing data. Are the missing data in discharge observations, for example, the difference between 180 and 145? Where are the numerators and denominators?
**Response:** We have adjusted how we report missing data. Thanks for pointing out these issues.

**Comment:**  Thank you for further describing your informed consent practices.
**Response:** Thank you for this suggestion to describe informed consent practices. This has definitely strengthened the methods.


**RESULTS:**
**Comment:** If continuing to reference the separate surveys, would it be possible to include a table that summarizes which pieces of which survey were categorized into which domain for analysis, along with the scores in each? You present a lot of this data, but it’s hard to follow because it’s very complex and there are so many categories.
**Response:** Thank you for this helpful suggestion. While we do not continue referencing the individual surveys in the Results section, we do organize findings by participant group, which aligns with the original surveys. To improve clarity, we refer readers to Figure 1, where the legend shows which survey corresponds to each participant group. We also moved Table S1 into the main manuscript (now Table 2) to more clearly summarize the domain structure, including all items contributing to each domain score.

**Comment:**  Presentation of proportions and use of N vs n continues to be unclear, and nearly uninterpretable without very close reading or referencing your figures. Examples and suggestions to further simplify this for the reader:

1. When presenting a proportion, I don't think there's any need to present it as "n=," because you're not presenting the number, but the proportion. For example, "most caregivers (n=57/145; 39%) can more clearly be presented as "39% (57/145) of caregivers interviewed."

 2. For three categories on your flow chart, N is variably the total population of 180 discharge observations or interviews, vs the number of patients analysed UNDER each variable (sex, discharge status, discharge diagnosis, travel time, etc), with n representing all subgroups in all analyses, and the capitalization is not used uniformly in the manuscript (N vs n, example line 211 where n does not match the N used in the table to reference the same population). Interpreting which N/n you’re talking about is very difficult without referencing the chart. On line 210, n references a percentage! May consider using subscripts (Nrespondents vs Ntotal, for example, or defining different variable letters for better clarity.

 Why are all the Ns for the discharge characteristics 145 when you have 180 observations? Is this just a coincidence, or were that actually only 145 analyzable surveys? So is 145 your actual analyzed population, not the 180 observations? It becomes clear later that the 180 observations yielded other important data, but it’s not clear here where you present your demographic results that that is why the 180 number is important.
**Response:** We have adjusted how we report numbers. Hopefully this adds the necessary clarity.

 **Comment:** As mentioned in a prior comment, I recommend avoiding “trended toward” when discussing results that were not statistically significant - eg line 239. Your IQRs are the same, even if the medians are different, and your p-value is nonsignificant, so your data is not supportive of a difference.
**Response:** We agree and have removed this statement.

**Comment:**  Where are the admission care practice score integrated into your analysis of discharge readiness? It does not seem to be integrated into your stated primary or secondary outcomes, and all of the results you mention are in supplementary tables and figures - are they really critical to present here or should they be analyzed and written up separately? This is very interesting information worthy of reporting in the literature, perhaps in the context of looking at correlation between admission practices and discharge practices, but is it relevant to your outcome measures here?
**Response:** Thank you for this thoughtful comment. We agree that the admission care practice scores offer valuable context, particularly as elements like socioeconomic and medical history taking are highly relevant to discharge care practices. While not specified as a secondary outcome, we felt it was important to include these findings (presented on Pg 17) to support interpretation of discharge readiness. Unfortunately, we do not have sufficient data on admission practices alone to justify a separate manuscript at this time.

**Comment:**  Figure 3 is excellent and very clear! This is the place where I best understand your findings.
**Response:** Thank you. We appreciate the positive feedback.

 **DISCUSSION:**

**Comment:** I think the discussion is quite strong. Of note, I don't think I see the admission care practices reflected anywhere in it, which to me feels like another argument to describe those in a separate venue, or else incorporate them more thoroughly into your outcomes.
**Response:** Thank you for this insightful comment. We do consider admission care practices relevant to the study; however, as noted previously, we do not have sufficient data to develop a separate manuscript on this topic. Given reviewer feedback to shorten the manuscript, we chose to limit discussion of admission practices here to maintain focus on discharge outcomes.

**Comment:**  Thank you for addressing the national representativeness of the sample in your limitations; this adds strength and transparency to the paper in my opinion.
**Response:** Thank you for this feedback. We definitely agree it adds strength and transparency.


**Some copyedits:**
**Comment:**  Standardize under-5 or “under 5” mortality. “Under-5” is standard when the phrase is used as an adjective; when it is phrased as “children under five,” there is no hyphen.
**Response:** Thank you. This has been corrected throughout the manuscript.

**Comment:** Spell out all non-decimal numbers under 10. (see line 90)
**Response:**Thank you. This has been corrected throughout the manuscript.

**Comment:** “For-profit” and “not-for-profit” should be hyphenated.
**Response:** Thank you for catching this error. It has now been corrected throughout.

**Comment:**  Define the acronym PFNFP on first use of the phrase on line 86.
**Response:**This is corrected.

**Comment:**  Period missing line 87.
**Response:** Corrected, thank you.

**Comment:**  Health care should be two words in AMA style.
**Response:** Corrected to AMA style, thank you.

**Comment:** In-patient should be inpatient - line 100 and throughout.
**Response:** Corrected to inpatient throughout.

 **Figures and Tables:
Comment:** These are very nice and where your main argument is made most effectively, even with the difficulties noted about the flow chart.
**Response:** Thank you for the positive feedback.

7. PLOS authors have the option to publish the peer review history of their article ([what does this mean?](https://track.editorialmanager.com/CL0/https:%2F%2Fjournals.plos.org%2Fglobalpublichealth%2Fs%2Feditorial-and-peer-review-process%23loc-peer-review-history/1/010f01961011fb91-209bac16-c7b3-40c6-8daf-1b53d06d137d-000000/hi5mR4t-v_aNyb8V50fsrGnmZJDoes0xZRXJSVy1HIY=205)). If published, this will include your full peer review and any attached files.


 **Do you want your identity to be public for this peer review?** If you choose “no”, your identity will remain anonymous but your review may still be made public.


 For information about this choice, including consent withdrawal, please see our [Privacy Policy](https://track.editorialmanager.com/CL0/https:%2F%2Fwww.plos.org%2Fprivacy-policy/1/010f01961011fb91-209bac16-c7b3-40c6-8daf-1b53d06d137d-000000/Q7QSPsiyWPxBqzwLuv0KDmnz8Fbt0kxv1Z4bdEoyuEI=205).

Reviewer #1: No

Reviewer #2: No
