# Supplementary material for: Discharge policies and care practices for children with suspected sepsis: A health facility scan at a nationally representative sample of hospitals and health centres in Uganda
Source: PLOS Glob Public Health. 2025 Aug 22;5(8):e0003559. doi: 10.1371/journal.pgph.0003559 (PMC12373204; doi:10.1371/journal.pgph.0003559)
Supplement: Attachment — Submitted filename: Response to reviewers.docx [file pgph.0003559.s005.docx]

Reviewer #1:

1. This manuscript assesses the readiness of health facilities in Uganda to manage pediatric discharge care for children under 5 years or age, focusing on 36 representative health facilities. The authors have made significant revisions in response to reviewer comments, especially clarifying the primary and secondary outcomes, which have strengthened the manuscript.

Thank you

2. Due to its scope and design, as well as its potential to impact practices in LMICs, it continues to be a strong candidate for publication. A few areas would benefit from additional clarification and consistency.

Thank you

**Methods:**

3. The methodology for deriving the second primary outcome needs further clarification. Specifically, it is unclear how the five key discharge practices were calculated as binary variables when they are derived from seven domains. Please clarify whether a certain number of items from each domain needed to be present for a practice to be considered “present”, or whether the five key practices were predefined as binary outcomes with Scan 3 subsequently assessing greater detail within three of them, or another method. An explicit description would help improve transparency for the reader.

For the second primary outcome of the presence of absence of 5 key discharge practices, these were simply calculates as “done” or “not done”, hence their binary nature. We state (line 168-171) the following:

*Each of these five variables (post-discharge counselling, provision of take-home materials, post-discharge risk assessment, post-discharge referrals, and clinical assessment on the day of discharge) were binary variables, assessed as either present or absent.*

For this outcome we did not assess the topics presented for counselling, the types of materials provided, the type of risk assessment done, etc. but rather just reported whether anything at all was done. With regards to the seven domains, these 5 practices were merely components of these seven domains, nothing more. That is, these specific practices were found within these domains.

To add clarity, we have added in 1 sentence after the statement on these key practices being binary. We now state (line 171-173) that:

*“For the purposes of the primary outcome, no consideration was given to the content of these practices (ex. counselling topics, method of risk assessment, etc).”*

4. What components constitute a post-discharge risk assessment? A concise definition can help readers interpret this outcome.

Risk assessment relied on the health workers opinion rather than an objective risk assessment tool or algorithm. This was mentioned in the discission (Line 276-277). We have now specified that these were “*subjectively defined*” on line 145-146 of the methods.

5. Please clarify in the methods how these two secondary outcomes were assessed: (1) discharge-relevant admission practices, and (2) caregiver and health worker satisfaction. Were Scans 4 and 5 used for caregiver and health worker satisfaction, respectively? If so, please state this.

The supplement clearly outlines the specific items included in the discharge relevant admission practices. The results (line 296) refers to the supplement on this. Specifically, Table S2 specifies 42 items that were assessed. Table S4 specifically identifies the items in the caregiver and healthworker satisfaction survey (see line 316 and 325).

With regard to survey #4 and survey #5, you are correct in assuming these were for caregivers and healthworkers, respectively. We have clarified which survey is for which part in the methods (line 97-103).

6. The discharge-relevant admission practice outcome is not clearly described in the Methods section and is not integrated into the Discussion. Additionally, based on Table S2 and Figures S2a-e, some components of this outcome (for example, medical history items) are unrelated to discharge care, although it is true that discharge preparation begins at admission. The authors can consider removing this secondary outcome and associated supplementary tables/figures to streamline the manuscript and focus on the many other findings already described, or clearly incorporate it in the Methods and Discussion section.

We believe that medical history items are critical to discharge. For example, many of these items have been robustly proven to be related to post-discharge mortality, including abnormal vital signs, maternal education, feeding, access to clean water, and many others. Please see:

<https://www.thelancet.com/journals/lanchi/article/PIIS2352-4642(23)00052-4/abstract>

and

<https://www.thelancet.com/journals/eclinm/article/PIIS2589-5370(23)00557-6/fulltext>

Indeed, it is these admission features that we have used to build prediction models that identify the “at risk” child.

Please see: <https://journals.plos.org/globalpublichealth/article?id=10.1371/journal.pgph.0003050>

If at the point of discharge, the discharging health worker is unable to understand and see the context of the illness from the point of admission, he/she will not be able to properly understand the risk of the child during the recovery period. Separating discharge from admission conceptually is indeed something that we are very keen to eliminate, as this vertical approach is part of the problem, and relegating all discussion of this to another paper further obscures the critical nature of this issue. While we understand that a comprehensive overview of all those pieces relevant to discharge may provide helpful to readers, we do not believe that a single paper can accomplish all of these tasks. This paper is already quite long and based on prior reviews we have already been forced to remove our qualitative analysis based on the healthworker and caregiver feedback from survey 4 and 5. Our research group is building an evidence base to be used in advancing discharge and post-discharge care in settings like those seen here. One single paper cannot accomplish this, but as you have pointed out, there are key messages in this paper that must be disseminated in the medical literature. We can assure you that many other angles are being addressed when it comes to post-discharge mortality in these settings.

7. Please ensure that the categories of the second primary outcome and secondary outcomes are listed in the same order across the abstract, methods, results, discussion, and tables for reader comprehension.

Thank you for pointing this out. We have updated the abstract to reflect the order form the main text of the manuscript. A few pieces in the results require the order to be different since the order reflects frequence, which we believe is a better way to report results.

**Results:**

8. Please indicate how many hospitals and health centres are represented in each of the PNFP and public categories. This could be added after the first sentence in the Results (Line 206) and listed in Table 1 under the respective headings.

Done

9. Lines 224-241, lines 259-277, (and lines 291-310 if keeping in the manuscript): The number of statistical comparisons make interpretation difficult. Consider highlighting only the most relevant findings in the narrative and grouping results by domain in each of the outcomes (for example, for resource availability grouping by infrastructure, equipment, etc). If possible and where relevant, consolidate comparisons between hospitals and health centres and between PNFP and public facilities to improve readability.

Thank you for this suggestion. We have carefully re-read these sections and edited these to add clarity. Several statistical comparisons have been removed, and furthermore a focus on overall performance as opposed to comparisons has been emphasized. We agree that much of this removed content is easily viewed in the tables and is not necessary in such detail throughout these sections.

10. Line 236: The phrase “though most facilities had key equipment available to assess post-discharge vulnerability” is vague and potentially subjective. Consider removing or rephrasing, as definitions of “key” can vary.

Done

11. Line 272-273: Please include the numbers or p-values to support the statement that discharge forms were more complete at health centres.

Done

12. Line 278: Consider beginning this section with a new subheading, as it introduces a secondary outcome of reported vs observed discharge practices.

Though we understand that the findings related to the reported vs observed key practices is somewhat separate from the key practices themselves, we do believe this fits thematically within the existing heading of “Key Discharge Practices”. We would prefer keeping this as is but will change if the editor feels that an additional sub-heading will be helpful to the reader.

13. Line 279: Please clarify why the fifth key discharge practice was not included in the reported vs observed comparison.

We agree that perhaps it should have been included, but the 5^th^ practice was doing a clinical assessment prior to discharge, and we (perhaps incorrectly) assumed this would be done considered a part of the reported practice in all sites. We would have been surprised to see any site reporting that a clinical assessment is not a part of their usual practice. However, we are now including this item for future assessments, as we agree that it would have been ideal if this was assessed in this manner.

**Discussion:**

14. First paragraph: Consider revising the opening paragraph of the Discussion to summarize the most important findings across all of the primary and secondary outcomes in order to frame the remainder of the discussion.

We have revised this to some degree. This paragraph does address the key highlights of the primary, and to a lesser extent, secondary outcomes. We now state (line 332-343):

*Our study assessed discharge related resources and care practices across hospitals and health centers, identifying key areas where improvement could help to address the high post-discharge mortality rate among children. Resource availability* *assessments revealed that hospitals were generally better equipped than health centres across all domains, except for Forms & Job Aids. There were no significant differences in resource domain scores between ownership types. All facilities reported notable gaps in availability of equipment required for optimal discharge care. This was evident in the inconsistent performance and documentation of physical measurements at both hospitals and health centres, even when resources were available. The use of simple tools to identify children at risk of poor post-discharge outcomes is increasingly being developed for use in similar settings [25–28]. Many features used in these models were routinely captured though the lack of pulse oximeters_,_ EMRs, and other technological resources highlights important barriers to scaling embedded processes which leverage such innovations.*

15. Line 347-348: Please revise this sentence to accurately reflect the findings in Table 2. Based on the data, health centres scored significantly higher in discharge counseling and discharge day clinical assessments, while hospitals scored significantly higher in the remaining three discharge care practice categories. The phrase “similar findings were also noted” may also need to be revised or removed, as the findings differ across categories.

We agree and have revised our discussion to reflect this, by combining this with the earlier paragraph. We now state (line 344-353):

*Our study revealed a lack of job aids across all facilities. Job aids are particularly valuable in settings with frequent understaffing and limited health worker expertise and training in discharge planning. For example, in this assessment only a single facility had a discharge policy, or a follow-up referral form, and none had counselling guides to help facilitate the discharge process. The absence of material such as these may contribute towards lower satisfaction with the discharge process among health workers [29,30] While resources undoubtedly represent a critical component necessary for the provision of quality care, many other factors, including leadership, good governance, and effective and continuous training also heavily influence care quality [29]. We found that despite fewer resources, health centres scored significantly higher in discharge counselling and discharge clinical assessments than hospitals.*

**Other:**

16. A thorough copy-editing is recommended to address minor spelling and grammatical errors. An few examples (non-exhaustive) are noted below.
    - Figure 1 title: Facility enrolment-> facility enrollment

Done

- Table 1: In the caption, remove “of resource availability.” The p-value for “facility has referral clinics that follow-up children post-discharge” should be marked with an asterisk for significance.

Thank you. These have been corrected.

- Line 215: “within a 1 hour” -> “within 1 hour.

Done

Overall, this manuscript provides important insights into pediatric discharge readiness in low and middle income countries and has the potential to make a valuable contribution to the literature.
